# Turnovers of Sex-Determining Mutation in the Golden Pompano and Related Species Provide Insights into Microevolution of Undifferentiated Sex Chromosome

**DOI:** 10.1093/gbe/evae037

**Published:** 2024-02-26

**Authors:** Liang Guo, Danilo Malara, Pietro Battaglia, Khor Waiho, D Allen Davis, Yu Deng, Zhongyuan Shen, Ke Rao

**Affiliations:** State Key Laboratory of Developmental Biology of Freshwater Fish, Hunan Normal University, Changsha, Hunan Province 410081, China; South China Sea Fisheries Research Institute, Chinese Academy of Fishery Sciences, Guangzhou, Guangdong Province 510300, China; Stazione Zoologica Anton Dohrn, Integrated Marine Ecology Department, CRIMAC, Calabria Marine Centre, Amendolara 87071, Italy; Integrated Marine Ecology Department, Stazione Zoologica Anton Dohrn, Sicily Marine Centre, Messina 98168, Italy; Higher Institution Centre of Excellence (HICoE), Institute of Tropical Aquaculture and Fisheries, Universiti Malaysia Terengganu, Kuala Nerus, Terengganu 21300, Malaysia; Centre for Chemical Biology, Universiti Sains Malaysia, Minden 11900, Malaysia; Department of Aquaculture, Faculty of Fisheries, Kasetsart University, Bangkok 10900, Thailand; School of Fisheries, Aquaculture and Aquatic Sciences, Auburn University, Auburn, AL, 36830, USA; State Key Laboratory of Developmental Biology of Freshwater Fish, Hunan Normal University, Changsha, Hunan Province 410081, China; State Key Laboratory of Developmental Biology of Freshwater Fish, Hunan Normal University, Changsha, Hunan Province 410081, China; State Key Laboratory of Developmental Biology of Freshwater Fish, Hunan Normal University, Changsha, Hunan Province 410081, China

**Keywords:** sex chromosome, alternative splicing, sex-determining mutation, zebrafish mutant, sex-differential selection, simulation

## Abstract

The suppression of recombination is considered a hallmark of sex chromosome evolution. However, previous research has identified undifferentiated sex chromosomes and sex determination by single SNP in the greater amberjack (*Seriola dumerili*). We observed the same phenomena in the golden pompano (*Trachinotus ovatus*) of the same family Carangidae and discovered a different sex-determining SNP within the same gene *Hsd17b1*. We propose an evolutionary model elucidating the turnover of sex-determining mutations by highlighting the contrasting dynamics between purifying selection, responsible for maintaining W-linked *Hsd17b1*, and neutral evolution, which drives Z-linked *Hsd17b1*. Additionally, sporadic loss-of-function mutations in W-linked *Hsd17b1* contribute to the conversion of W chromosomes into Z chromosomes. This model was directly supported by simulations, closely related species, and indirectly by zebrafish mutants. These findings shed new light on the early stages of sex chromosome evolution.

**Significance**

An intriguing phenomenon that has been observed is the absence of recombination suppression between sex chromosomes and the turnover of sex-determining mutations. By focusing on the turnover of these mutations, we shed light on this fascinating pattern.

## Introduction

The sex chromosomes of birds and mammals exhibit a high degree of differentiation (Cortez et al. 2014; Xu et al. 2019), whereas many lineages of fish, amphibians, and nonavian reptiles possess only slightly differentiated sex chromosomes (Vicoso 2019). According to the canonical theory, sex chromosomes have repeatedly and independently evolved from autosomes when one of the sex chromosomes acquires a sex-determining locus (Muller 1918; Beukeboom and Perrin 2014). Regardless of whether the sex chromosomes are young or degenerated, recombination suppression has evolved convergently in a ubiquitous pattern (Ponnikas et al. 2018). The most widely cited explanation is sexually antagonistic selection, which was proposed by Fisher (1931) and further developed theoretically by Charlesworth and Charlesworth (1980), Bull (1983) and Rice (1987). However, alternative hypotheses such as meiotic drive (Úbeda Patten and Wild 2015), heterozygote advantage (Charlesworth and Wall 1999), and genetic drift (Lande 1985; Jeffries et al. 2021) have also been proposed. Regrettably, as pointed out by Wright et al. (2016) there exists insufficient empirical evidence to validate or disprove these hypotheses. One significant obstacle in conducting empirical research on sex chromosome evolution lies in the fact that many investigated species have preexisting differentiated sex chromosomes with established nonrecombination regions. The difficulty in distinguishing between causes and consequences of recombination suppression underscores the importance of examining evidence from sex chromosomes at earlier stages of divergence, which is crucial for gaining a more comprehensive understanding of the initial process and underlying causes of recombination suppression.

Numerous poikilothermic organisms exhibit homomorphic sex chromosomes, and the rapid advancements in sequencing technologies, particularly long-read sequencing technology, facilitate the study of sex chromosomes. However, progress in unraveling the mechanisms underlying recombination suppression remains limited due to the already-differentiated state of sex chromosomes in many heteromorphic model systems. In contrast, undifferentiated sex chromosomes have been observed in the greater amberjack (*Seriola dumerili*) (Koyama et al. 2019) and the tiger pufferfish (*Takifugu rubripes*) (Kamiya et al. 2012). In these two species, sex is determined by a single sex-determining mutation, i.e. SNP1196 within the *Hsd17b1* gene and SNP7271 within the *Amhr2* gene, respectively, and there is no divergence between proto-sex chromosomes beyond the sex-determining mutations. The tiger pufferfish has shared its sex-determining gene and sex-determining mutation with at least eight congeneric species for 5 million years (Kamiya et al. 2012; Kabir et al. 2022), while the greater amberjack shares the same with at least two congeneric species for 29 to 55 million years (Koyama et al. 2019). However, the mechanisms responsible for maintaining undifferentiated sex chromosomes in these species require further investigation. Such investigation would also provide insight into the establishment of recombination suppression and nonrecombination regions.

In this study, our aim was to investigate the evolutionary trajectory of undifferentiated sex chromosomes in the golden pompano (*Trachinotus ovatus*) and other closely related species. The golden pompano and the greater amberjack belong to subfamilies Naucratinae and Trachinotinae, respectively, both nested under the family Carangidae (Santini and Carnevale 2015; Damerau et al. 2018). Through a whole genome-wide analysis, we identified a sex-determining mutation, Chr16:g.18219150A > G located within the first intron donor splice site (GT-AG) of *Hsd17b1*, that is exclusively associated with the phenotypic sex in the golden pompano. Interestingly, no sequence divergence was observed beyond the sex-determining SNP. Based on these findings, we propose a microevolutionary model to comprehend the turnover of sex-determining mutations. The turnovers of sex-determining mutations in the closely related species and the manifestation of male phenotype in homozygous mutant zebrafish support our model. We also conducted time-forward simulations to investigate the evolutionary forces underlying undifferentiated sex chromosomes and turnovers of sex-determining mutations.

## Results

### The Monogenic Sex-Determination System is Supported by Evidence From Sex Ratio and Linkage Mapping

**Fig. 1.**
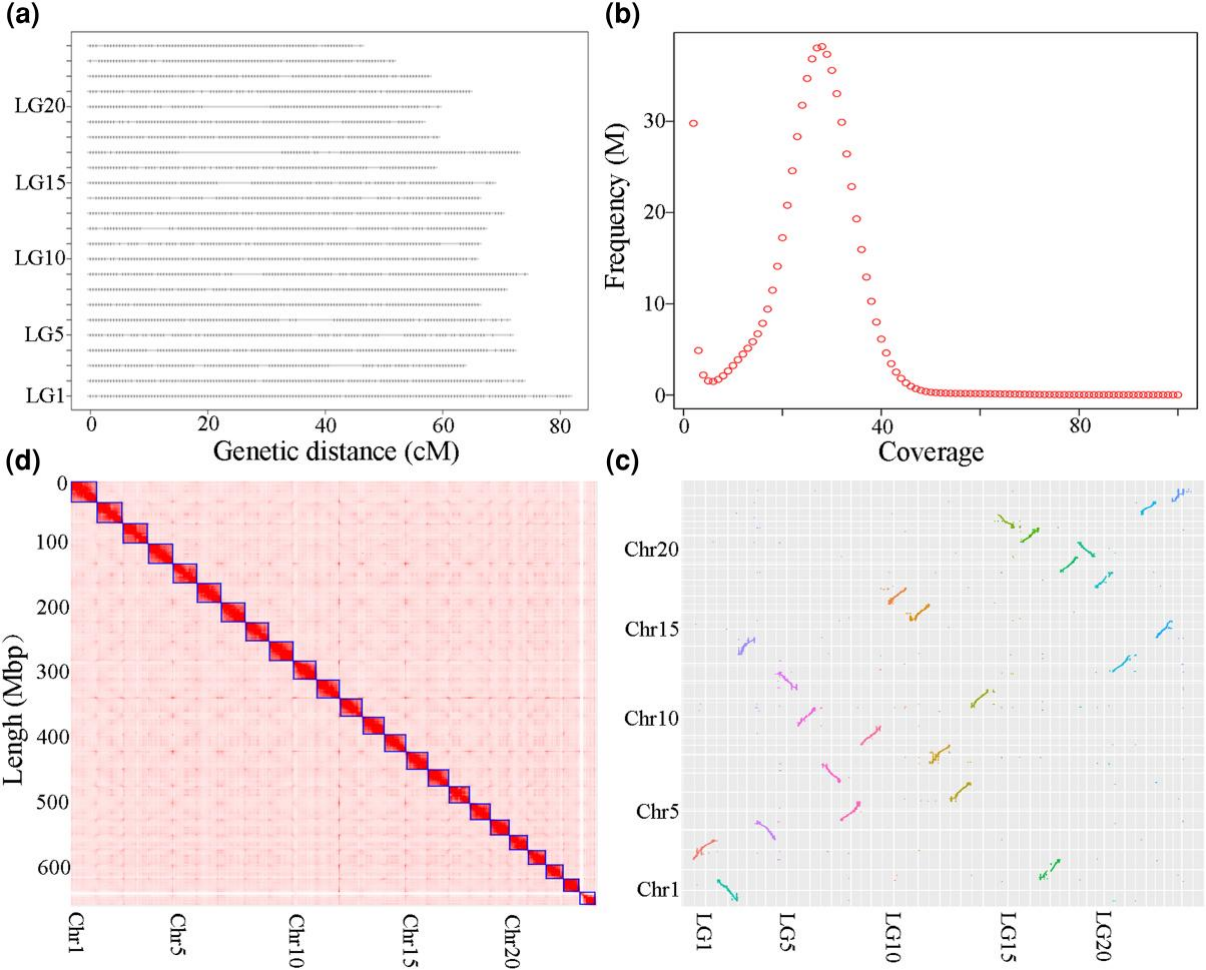
The chromosome-level genome assembly of the golden pompano. a) Genetic linkage map constructed with a full-sib family (F201803). b) K-mer profile. c) Heatmap of long-range interactions of the chromosomes captured by Hi-C sequencing. d) Comparison of the genome assembly and the genetic linkage map.

To trace the sex-determining system in the Carangidae family, we conducted a study on the sex ratio of golden pompano families and performed quantitative trait locus mapping (QTL-mapping) to identify its sex determining loci. A pedigree consisting of five full-sibling families was previously constructed and marked with passive integrated transponders (Guo et al. 2020). The phenotypic sex of 1,006 progeny was successfully identified through gonad observation, including 502 females and 504 males (supplementary table S1, Supplementary Material online). The sex ratio within each full-sibling family was approximately 1:1 (*P-*value = 0.74, chi-square test), indicating that a single genetic factor controls sex determination in the golden pompano. Previous studies have also reported sexual size dimorphism in this species (Sun et al. 2022). In our study, we observed significant differences in body weight among sexes (*P-*value < 0.001, analysis of variance) and families (*P-*value < 0.001, analysis of variance). Additionally, utilizing SNP-calling data from family F201803 consisting of two parents and 100 progeny against genome assembly (GCA_900231065.1), we identified 24 linkage groups (Fig. 1a). The sex-averaged genetic map covered a distance of 1984.40 cM, comprising 3,307 segregating sites and 689,070 SNPs. A single significant sex QTL with an LOD threshold score of 4.4 was identified, explaining 50.3% of the phenotypic variance. This QTL spanned from 38.00 to 66.01 cM on LG11 with a peak at 53.50 cM. Multiple QTL mappings confirmed the presence of only one QTL in this interval. In summary, our findings suggest that a single gene is responsible for sex determination in the golden pompano.

### Constructing a High-Quality Genome Assembly for the Golden Pompano

A high-quality genome assembly is a prerequisite for elucidating the divergence of sex chromosomes. Hi-C sequencing assessment revealed numerous misplaced contigs in the previous genome assembly (GCA_900231065.1) (Guo et al. 2021). To further evaluate the sex chromosome of this previous assembly, we conducted a family-based genome-wide association study (GWAS) using data from family F201803. Surprisingly, we identified five significant peaks on distinct pseudochromosomes (supplementary fig. S1, Supplementary Material online), which contradicted the notion that sex is controlled by a single factor, and also suggests potential misassembly of the golden pompano's sex chromosome in the previous assembly. To tackle this issue, we reconstructed a genome assembly utilizing 142× PacBio long reads from a male individual with low heterozygosity (Fig. 1b). The primary assembled contigs were contiguous with a N50 of 23.14 Mb (supplementary tables S2 to S4, Supplementary Material online). Subsequently, the contigs were scaffolded using 179× Hi-C paired-end reads from Zhang et al. (2019). The resulting chromosome-level assembly (GCA_022709315.1) comprised of 24 pseudochromosomes, totaling up to 662.70 Mb (supplementary fig. S2 and supplementary tables S5 to S8, Supplementary Material online and Fig. 1c). Remarkably, a perfect one-to-one correspondence was observed between the linkage groups and the pseudochromosomes, with an average Pearson correlation coefficient of 0.98 (Fig. 1d and supplementary fig. S3, Supplementary Material online). A Benchmarking Universal Single-Copy Orthologs (BUSCO) search against the 4,584 single-copy orthologs for Actinopterygii indicated that only 2.2% of core genes were absent from this assembly. Additionally, this assembly had a mapping rate of 99.57% for Illumina genome sequencing reads. Overall, our findings demonstrate that the newly reconstructed genome assembly provides a reliable foundation for future investigations into the sex determination system of the golden pompano. We predicted 25,720 protein-coding genes and estimated the divergence time between the golden pompano and the greater amberjack to be approximately 57.45 million years ago (95% confidence interval: 40.82 to 74.32 million years ago) (supplementary fig. S4, Supplementary Material online).

### Validating the Sex-Determining SNP that is Exclusively Associated through GWAS

**Fig. 2.**
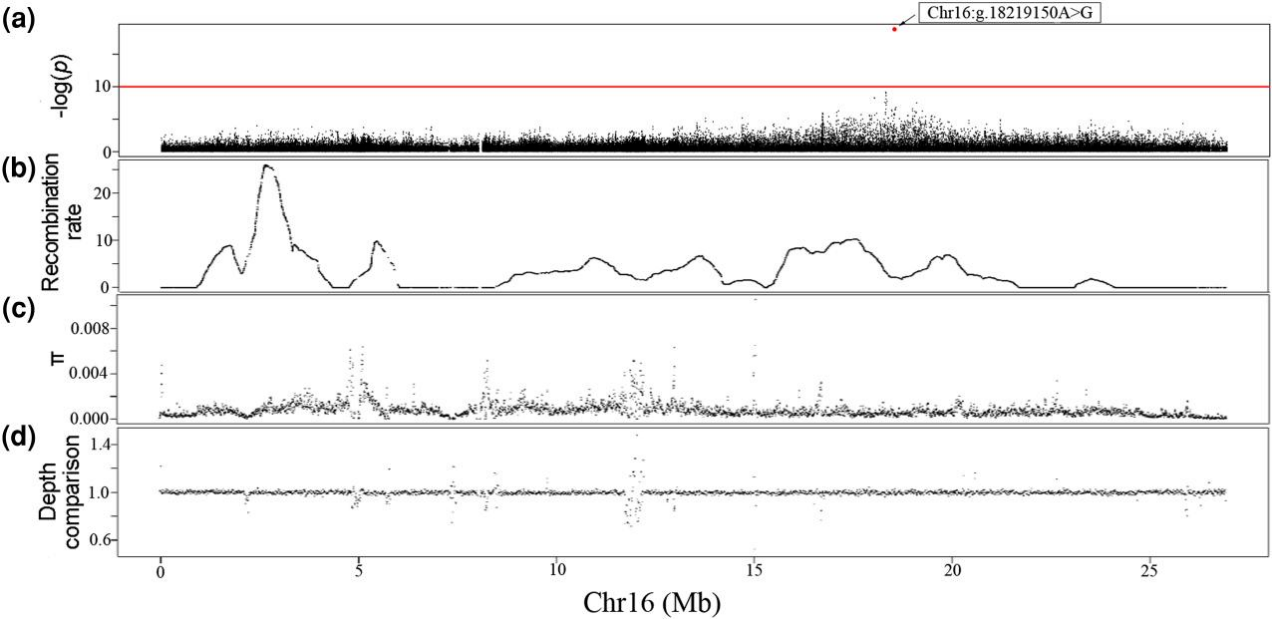
Sex chromosome statistics. a) Manhattan plot of the GWAS of sex. The highlighted point indicates the perfectly associated SNP (Chr16:g.18219150A > G). b) The recombination rate of the female parent in the mapping family (F201803). c) Nucleotide diversity (nonoverlapping windows, window size = 10 kb) in the natural population. d) Comparison of the depth of coverage (nonoverlapping windows, window size = 10 kb) in the parents of the full-sib family (F201803).

A GWAS on a natural population in golden pompano was conducted to pinpoint the specific mutation responsible for its sex determination. Our results (Fig. 2a and supplementary fig. S5, Supplementary Material online) revealed a single site, Chr16:g.18219150A > G, was significantly associated with sex determination (*P*-value of 1.36 × 10^−19^, 37 males and 45 females). This SNP displays a female heterogametic sex-determining system, where all females exhibit heterozygote (G/A), while all males are homozygote (A/A). Additionally, the second most significant site was Chr16:g.17999797C > T with a *P*-value of 6.91 × 10^−10^. Our findings suggest that Chr16:g.18219150A > G serves as the key mutation responsible for sex determination in golden pompano.

### Confirmation of Alternative Splicing Events in *Hsd17b1* Gene Expression within Testis and Ovary

**Fig. 3.**
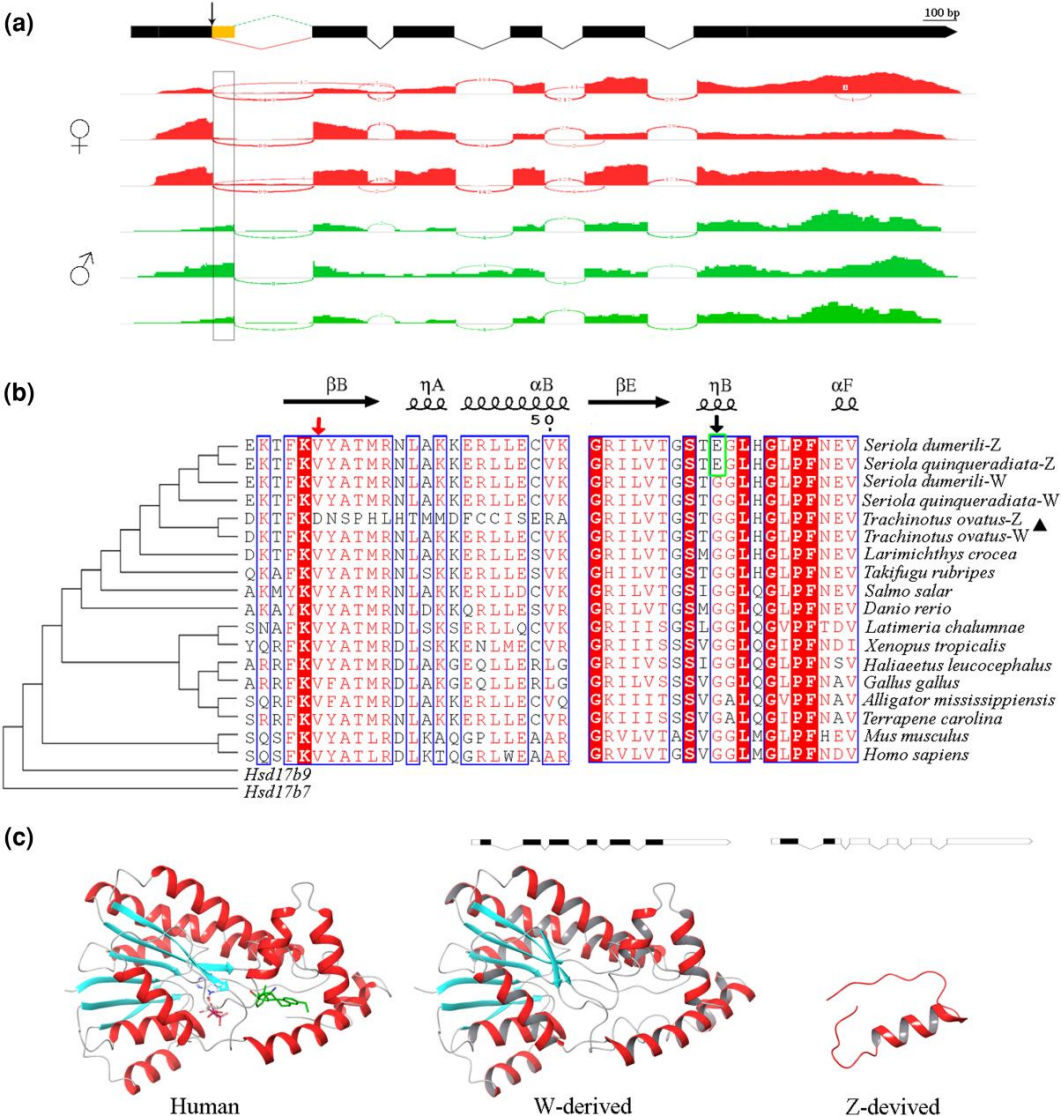
Alternative splicing of the *Hsd17b1* gene in the golden pompano. a) Schematic diagram of the gene *Hsd17b1* and Sashimi plot of its expression in ovaries and testes (*n* = 3). The W-derived transcript was highly expressed in ovaries, and the Z-derived transcript, including extra 64 bp at the end of the first exon, was mainly expressed in the testis. The arrows indicate sex-determining mutations, Chr16:g.18219150A > G. b) Multiple sequence alignments. The arrows indicate sex-determining mutations in the golden pompano and in the greater amberjack, respectively. The W-derived protein sequence of *Hsd17b1* observed in the golden pompano is conserved across vertebrates, including humans and coelacanths, while the alternative 5′ splice site selection of the first intron incorporates additional 64 nucleotides in the Z-derived transcript, resulting in a shift in coding frame and the introduction of a premature termination codon. The sex-determining mutation in the Z-derived protein of the greater amberjack breaks the ηB helix, significantly impairs the conversion activity from estrone to estradiol and finally leads to binary sex development (Koyama et al. 2019). c) Three-dimensional models of the human protein crystal structure (ID: 1iol.1), predicted W-derived protein (290 aa) and predicted Z-derived protein (87 aa). The cofactor NADPH (red sticks) and the catalysate estradiol (blue sticks) are also included in the human crystal protein.

Further analysis of *Hsd17b1* in teleost revealed that the SNP Chr16:g.18219150A > G was located at the alternative splice donor site (GT-AG) of the first intron (Fig. 3 and Supplementary fig. S6, Supplementary Material online). PacBio Iso-seq data obtained from a 1-yr-old ovary of the golden pompano demonstrated that two transcripts (W/Z-derived) were expressed from the *Hsd17b1* gene. Illumina RNA-seq (supplementary table S20, Supplementary Material online) confirmed that ovarian tissues primarily expressed the W-derived transcript and showed less expression of the Z-derived transcript. In contrast, testicular tissues exclusively expressed the transcript derived from Z chromosome (Fig. 3a). The presence of these two transcripts in developing ovaries and testes of 1-yr-old golden pompano was confirmed by Sanger sequencing (supplementary fig. S7, Supplementary Material online). The comparison between the two transcripts revealed that alternative 5′ splice site selection of the first intron incorporates additional 64 nucleotides in the Z-derived transcript, resulting in a shift in coding frame and the introduction of a premature termination codon (Fig. 3a).

### Presence of Undifferentiated Sex Chromosomes in the Golden Pompano

The W and Z chromosomes were compared to validate the hypothesis that there is no differentiation in sex chromosomes of golden pompano. The results demonstrate that depths of coverage and nucleotide diversity between sexes were nearly identical (Fig. 2). Furthermore, there was no indication of high linkage disequilibrium (*r*^2^ > 0.8) within the *Hsd17b1* gene (supplementary fig. S8, Supplementary Material online). Additionally, the recombination rate and depth difference between sexes around Chr16:g.18219150A > G were comparable to those observed in other regions. (supplementary figs. S9 and S10, Supplementary Material online). These findings suggest that, apart from the sex-determining SNP, the sex chromosomes were undifferentiated.

### Macroevolutionary Analysis Reveals the Presence of Purifying Selection Acting on the *Hsd17b1* Gene

To explore the macroevolution of *Hsd17b1* in vertebrates, we retrieved and compared its coding sequences from fish, amphibians, birds, and mammals. Codon-based tests of neutrality indicated that the nonsynonymous substitution rate of *Hsd17b1* was significantly lower than its synonymous substitution rate (d*N* − d*S* < 0, *P* < 0.01, as presented in supplementary tables S21 and S22, Supplementary Material online). This suggests that the coding sequence of *Hsd17b1* has primarily experienced purifying selection. We have also observed that alternative 5′ splice site selection in the Z-derived *Hsd17b1* of the golden pompano interrupts a conserved β-sheet across vertebrates (Fig. 3). Furthermore, we have identified that the ancestral allele of the sex-determining SNP is allele G, while allele A represents a derived variant.

### Refining a Microevolutionary Framework for *Hsd17b1*

The function of HSD17B1 derived from W/Z chromosome has been investigated in the greater amberjack, revealing that Z-derived HSD17B1 is dysfunctional while W-derived HSD17B1 remains functional (Koyama et al. 2019). Based on our findings, we propose a microevolutionary model of *Hsd17b1* in both golden pompano and greater amberjack involving, (1) purifying selection of W-linked *Hsd17b1*, (2) neutral evolution of Z-linked *Hsd17b1*, and (3) loss-of-function mutations leading to the transformation of W chromosome to the Z chromosome.

### Rephrasing the Impact of Evolutionary Forces on Sex-Determining Mutation Turnover

**Fig. 4.**
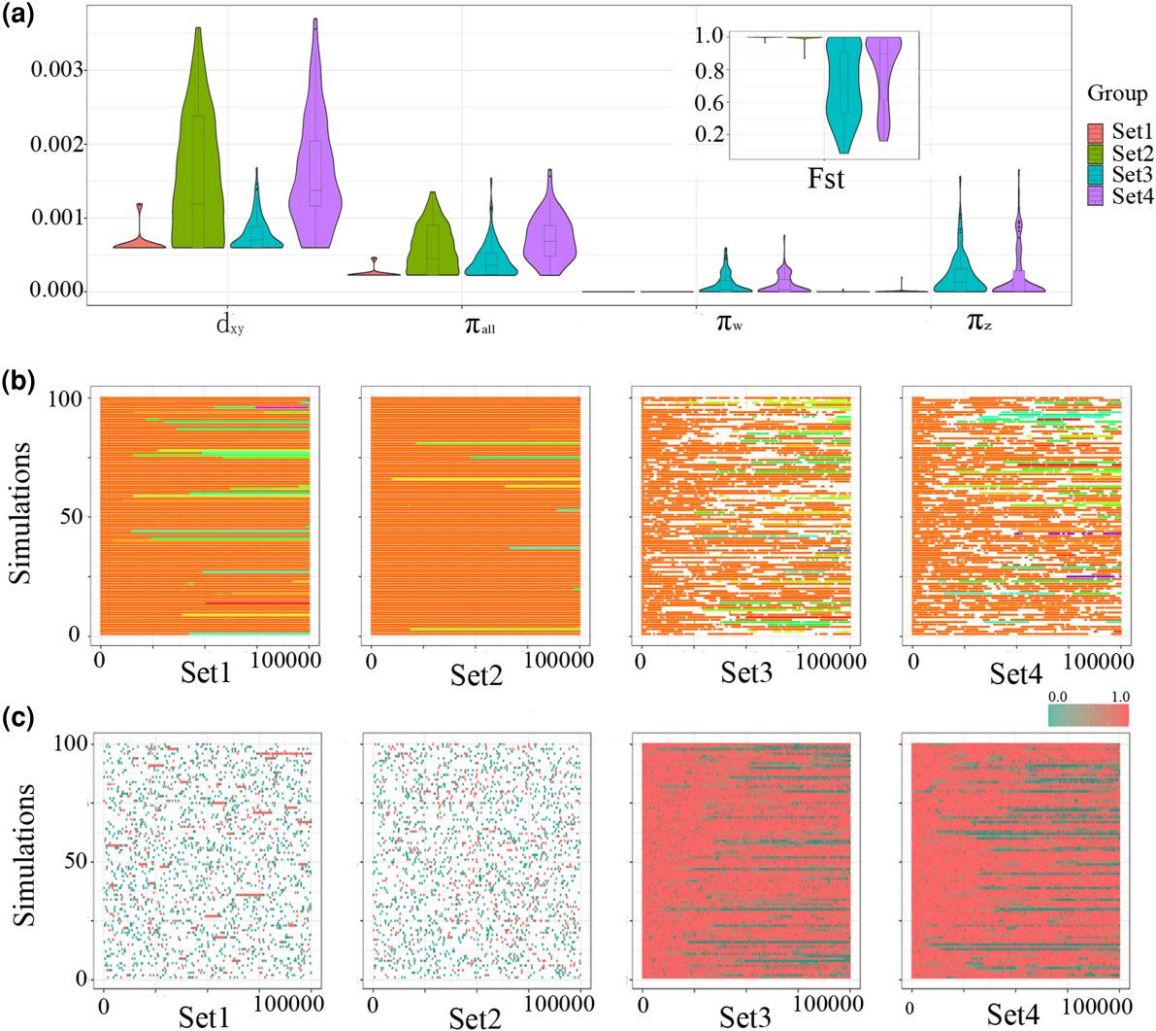
The simulations of *Hsd17b1* microevolution. The simulations were performed for 100,000 generations with four sets of parameters. Set1: *μ* = 1 × 10^−8^, *r*_0_ = 1 × 10^−6^, 2*N* = 100; Set2: *μ* = 1 × 10^−8^, *r*_0_ = 1 × 10^−8^, 2*N* = 100; Set3: *μ* = 1 × 10^−8^, *r*_0_ = 1 × 10^−6^, 2*N* = 10,000; Set4: *μ* = 1 × 10^−8^, *r*_0_ = 1 × 10^−8^, 2*N* = 10,000. For each set of parameters, 100 replicated simulations were conducted. a) The divergence at the end of the simulations. b) The fixed sex-determining mutations. The sex-determining mutations were shown in different colors. c) The nonfixed sex-determining mutations. The color indicates the frequency of the loss-of-function mutations, with red indicating high frequency and green indicating low frequency.

We conducted forward-time simulations based on Wright–Fisher model to investigate the prevalence and influencing factors of a single sex-determining mutation within a population, while considering recombination rate and population size (Set1 to Set4, Table 1, Fig. 4). Four parameter sets were defined with recombination rates of 1 × 10^−6^ or 1 × 10^−8^, and population sizes (2*N*) of either 100 or 10,000. The simulations were conducted for 100,000 generations and replicated 100 times for each parameter set. The initial state consisted of only one mutation in Z-linked gene sequence of *Hsd17b1*, specifically located at the alternative splicing donor site in the first intron. A turnover event was considered to have occurred when the final state had a different position as the sex-determining mutation without any other nonfixed or fixed sex-determining mutations present. After performing 100 replicated simulations using a population size of 100 (2*N*), we observed turnovers of sex-determining mutations in 26 simulations within Set1 and 11 simulations within Set2, correspondingly, at recombination rate of 1 × 10^−6^ and 1 × 10^−8^. Additionally, two or more fixed mutations were observed in three simulations with a recombination rate of 1 × 10^−6^ (Set1), while in 32 simulations with a recombination rate of 1 × 10^−8^ (Set2). We found that single loss-of-function mutations were responsible for determining sex in the majority of cases, they were observed in 97 (Set1) and 68 (Set2) simulations, respectively. We observed turnovers of sex-determining mutations in 15 and 12 simulations, respectively, with a recombination rate of 1 × 10^−6^ (Set3) and 1 × 10^−8^ (Set4) when the population size was 10,000 (2*N*). Interestingly, there were non-fixed sex-determining mutations in 63 and 63 simulations with recombination rate of 1 × 10^−6^ (Set3) and 1 × 10^−8^ (Set4), respectively. These results suggest that a small population size and a higher recombination rate contribute to the fixation of a single loss-of-function mutation determining sex. The low heterozygosity of the golden pompano (0.13%) compared to most fish species (supplementary table S23, Supplementary Material online) further supports the role of genetic drift in determining sex with a single mutation. The heterozygosity is 0.65% (Sarropoulou et al. 2017), and a nonfixed sex-determining mutation (SNP1195, ∼1%) has been observed in the greater amberjack population (Koyama et al. 2019), which is consistent with simulations of a larger population size. Our findings suggest that relatively higher recombination rate and genetic drift are critical factors in sex-determining mutation turnover.

**Table 1:** The parameters and results of the simulations

	*μ*	*r*_0_	2*N*	TS (%)	Simulations with # fixed sex-determining mutation					Simulations with # nonfixed sex-determining mutation					
					0	1	2	3	4	0	1	2	3	4	5
Set1	1E−8	1E−6	100	26	0	97	3	0	0	99	1	0	0	0	0
Set2	1E-8	1E-8	100	11	0	68	25	6	1	99	1	0	0	0	0
Set3	1E−8	1E−6	10,000	15	63	37	0	0	0	35	2	32	22	5	4
Set4	1E−8	1E−8	10,000	12	63	37	0	0	0	36	1	37	18	4	4

We conducted 100 replicated simulations for each combination of mutation rate (*μ*) and recombination rate (*r*_0_). By comparing the initial and final states, we documented the percentage of simulations where turnovers of the sex-determining locus occurred (TS). We also recorded whether the sex-determining mutations were fixed or nonfixed in the final state. Set1 exhibited greater consistency with actual conditions, encompassing parameters and turnovers of sex-determining mutations.

### Turnovers of Sex-Determining Mutations Among Closely Related Species

**Fig. 5.**
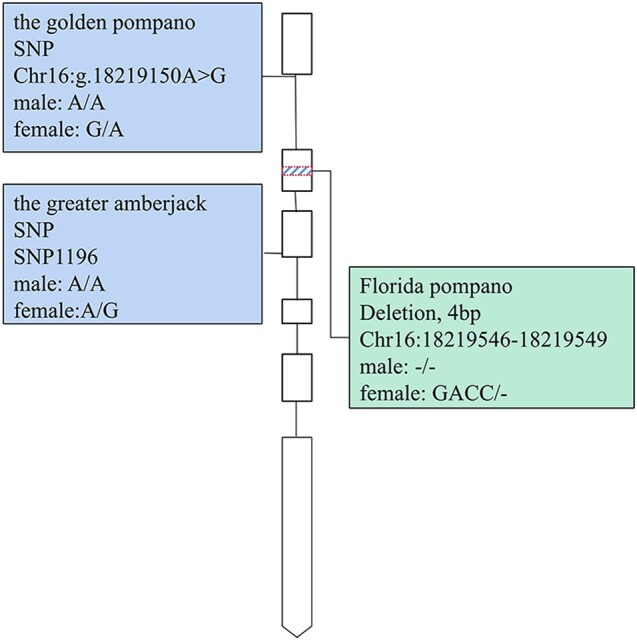
Turnovers of sex-determining mutation within the gene *Hsd17b1*.

As our proposed model predicted, closely related species may have additional turnovers of sex-determining mutations. To test this hypothesis, we collected 21 Florida pompano (*T. carolinus*) samples (7 males and 14 females) and subjected them to resequencing. The sex-determining mutations of the golden pompano and the greater amberjack were not detected (Fig. 5). Surprisingly, a 4-bp deletion from Chr16:18219546 to Chr16:18219549 in the second exon was found to be perfectly associated with sex (*P*-value = 4.5 × 10^−6^), with females having GACC/− and males having −/− (supplementary fig. S11, Supplementary Material online). Furthermore, there is no high level of linkage observed among the region around *Hsd17b1* gene in the population (supplementary fig. S12, Supplementary Material online).

### Knockout of *Hsd17b1* in Zebrafish Resulted in the Exclusive Production of Male Offspring

**Fig. 6.**
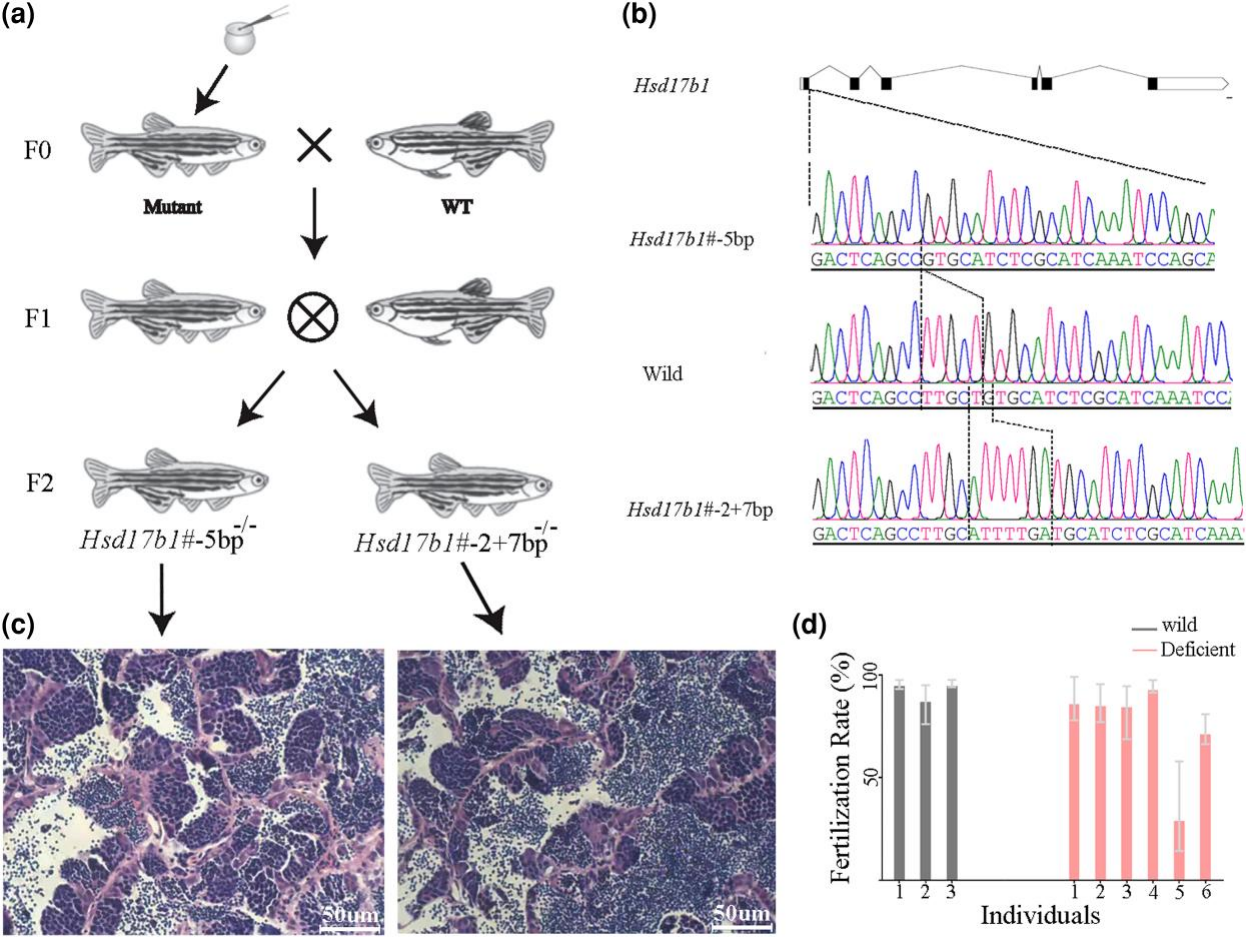
Establishment of *Hsd17b1* mutant lines in zebrafish. a) The strategy for establishment of mutation lines. b) Diagram of the mutant lines. c) Histological confirmation of the sex of homozygous mutant lines (*n* = 6, bar = 50 µm). All samples were collected at 145 d post-fertilization. d) The spawning ratio of wild males and mutant males crossed with wild-type females (*n* ≥ 3, mean with range).

To further investigate the sexually differential function of *Hsd17b1*, we generated two zebrafish lines with frameshift mutations located within the first exon (Fig. 6 and supplementary table S24, Supplementary Material online, *Hsd17b1*#-5bp and *Hsd17b1*#-2 + 7bp). Homozygous *Hsd17b1*-deficient individuals were exclusively males (20 individuals for line *Hsd17b1*#-5bp and 12 individuals for line *Hsd17b1*#-2 + 7bp), while heterozygous *Hsd17b1*-deficient individuals could develop into either females or males (2 males and 3 females for line *Hsd17b1*#-5bp, 2 females and 2 males for line *Hsd17b1*#-2 + 7bp). We have also observed a slight disparity in the fertilization rate between wild-type and homozygous *Hsd17b1*-deficient males (*P*-value = 0.047, Mann–Whitney test, *n* ≥ 3, supplementary table S25, Supplementary Material online). These findings offer further insight into the role of *Hsd17b1* in sex determination of fish.

## Discussion

In addition to tiger pufferfish and the greater amberjack, our research findings indicate that the golden pompano is another case with undifferentiated sex chromosomes. We proposed a novel model for better understanding the microevolutionary process of the sex-determining genes and mechanisms underlying maintenance of undifferentiated sex chromosomes as well as turnover sex-determining mutations.

### *Hsd17b1* is the Most Probable Candidate Gene for Sex Determination in the Golden Pompano

*Hsd17b1* has been identified as the most probable sex-determining gene in the greater amberjack (Koyama et al. 2019). The golden pompano and the greater amberjack are members of the same family but diverged approximately 57.45 million years ago. Our study provided further evidence for *Hsd17b1*'s involvement in sex determination, including balanced sex ratio across different families, discovery of single sex QTL, and an exclusive association between Chr16:g.18219150A > G and phenotypic sex. Notably, Chr16:g.18219150A > G is situated at an alternative splice donor site, leading to alternative splicing (Fig. 3a), which may result in the malfunction of the Z-derived protein HSD17B1. We propose that *Hsd17b1* functions in a dominant pattern in the golden pompano and the greater amberjack. As there is only one copy of *Hsd17b1* in the genome (supplementary fig. S13, Supplementary Material online), it is highly probable that the sex-determining gene *Hsd17b1* arose through allelic diversification.

*Hsd17b1* functions as a homodimer, catalyzing the interconversion between estrogens and androgens (Andersson 1995). In fish, estrogens are not only essential for ovarian differentiation but also maintenance (Guiguen et al. 2009; Li et al. 2019). The synthesis of estradiol from androstenedione in vivo follows the Δ4 (*Cyp19a1a* and *Hsd17b1*) or Δ5 (*Hsd17b3* and *Cyp19a1a*) pathway, which has been reported in several fishes (Tenugu et al. 2021). Our study demonstrated a male-only phenotype in zebrafish following *Hsd17b1* knockout. The consistent manifestation of male development with spontaneous mutants in the greater amberjack and the golden pompano, along with artificial mutation in zebrafish, suggests that the Δ4 pathway plays a crucial role during early sex differentiation and is conserved among teleosts. These findings demonstrate that *Hsd17b1* conservation is essential for female development in teleosts, and W-linked *Hsd17b1* is likely subjected to purifying selection.

The gene *Hsd17b1* in the greater amberjack and the golden pompano functions as a dominant factor. In the greater amberjack, a Z-linked missense mutation SNP1196 resulted in a 99.66% reduction of catalytic activity (Koyama et al. 2019). Meanwhile, in the golden pompano, a Z-linked allele A at the sex-determining SNP caused alternative splicing, reading frame shift, and an introduction of a premature termination codon. The deletions in Z-linked *Hsd17b1* in Florida pompano are likely to result in frameshift. Based on these findings, as well as the observation of exclusively male phenotype in homozygous mutants of *Hsd17b1* in zebrafish, it is probable that *Hsd17b1* is dispensable for male development and Z-linked *Hsd17b1* evolve nearly neutrally.

### What is the Rationale Behind the Presence of Undifferentiated Sex Chromosomes and the Occurrence of Turnovers in Sex-Determining Mutations?

The undifferentiated sex chromosomes have been maintained for 5 million years in *Takifugu* and 29 to 55 million years in *Seriola*, respectively (Kamiya et al. 2012; Koyama et al. 2019). Based on the findings of this study, it is likely that undifferentiated sex chromosomes have been preserved for 57 million years in the Carangidae family. Various hypotheses have been proposed to explain the adaptive or nonadaptive reasons for recombination suppression (Ponnikas et al. 2018; Jeffries et al. 2021). The widely accepted theory of sexually antagonistic selection posits that selection acts within a region between a sex-determining gene and a nearby locus with sex-specific effects, ultimately leading to recombination suppression and heteromorphic chromosomes (Fisher 1931; Rice 1987; Ponnikas et al. 2018). Recently introduced neutral model also offer alternative explanation for the evolution of suppressed recombination, suggesting that frequent neutral or nearly neutral mutations relative to recombination rates can lead to such suppression (Jeffries et al. 2021). However, the current body of evidence is insufficient to support these claims (Beukeboom and Perrin 2014; Ponnikas et al. 2018), and existing models fall short explaining long-term undifferentiated sex chromosomes, particularly with regard to sex-determining mutation turnovers.

It is important to note that these models do not comprehensively account for the sex-determining gene, including its sequence, origin, and function manner. As previously mentioned, the sex-determining gene *Hsd17b1* arose through allelic divergence and functions in a dominant manner. Our model demonstrates undifferentiated sex chromosomes with single sex-determining mutations occurring in 97% of cases and turnovers of sex-determining mutations happening in 26% of cases when the recombination rate, mutation rate, and population size were set at 1 × 10^−6^, 1 × 10^−8^, and 100, respectively. These results are consistent with our observations on the recombination rate, genetic drift, turnover of sex-determining mutation and the stability of sex chromosomes in both golden pompano and greater amberjack. The estimated local recombination rate flanking the *Hsd17b1* gene in golden pompano was two orders of magnitude higher than the average recombination rate observed in animals (Stapley et al. 2017). Meanwhile, the estimated heterozygosity was lower than most fish species (supplementary table S23, Supplementary Material online), indicating a small population size. Notably, perfect associations between loss-of-function deletions in Florida pompano with the phenotypic sex further validate the proposed model. According to the neutral model (Jeffries et al. 2021) and attrition model (Ellis et al. 1990), divergence is expected to accumulate when the recombination rate is of similar magnitude as the mutation rate or lower. We observed similar results in which 32% of replicated simulations exhibited two or more fixed sex-determining mutations when mutation rate, recombination rate and population size were set to 1 × 10^−8^, 1 × 10^−8^, and 100, respectively. In contrast, nonfixed sex-determining mutations co-existed in approximately 65% of replicated simulations when the population size was larger (2*N* = 10,000), regardless of whether the recombination rate was equal to or larger than the mutation rate. This discovery is in line with the presence of a low-frequency loss-of-function mutation SNP1195 (∼1%) within the greater amberjack population as documented by Koyama et al. (2019), and also the higher heterozygosity observed in the greater amberjack population compared to the golden pompano population. These findings suggest that the turnovers of sex-determining mutations and undifferentiated sex chromosomes in the golden pompano and the greater amberjack were contributed by strong genetic recombination and drift, as well as the functional manner of the sex determining gene of *Hsd17b1*.

We have observed that four master sex-determining genes, *gsdf-Y* in Medaka *Oryzias luzonensis* (Myosho et al. 2012), *sox3-Y* in *Oryzias dancena* (Takehana et al. 2014), *gdf6-Y* in killifish *Nothobranchius furzeri* (Reichwald et al. 2015), *gsdf-Y* in sablefish *Anoplopoma fimbria* (Herpin et al. 2021), have arose through allelic divergence and function by acquiring new spatial and temporal expression patterns. There genes are located on the early-stage sex chromosomes where additional mutations have accumulated. Obviously, these genes were responsible for male development in each species, while their X-linked counterparts also play indispensable roles in female development. Homozygous mutation of *gsdf* causes infertility in female Japanese medaka, zebrafish, and Nile Tilapia (Guan et al. 2017; Yan et al. 2017; Jiang et al. 2022). Loss-of-function of *sox3* leads to follicle development retardation and reduces fecundity in zebrafish (Hong et al. 2019). *gdf6* is a necessary factor in neurodevelopment (Gramann et al. 2019). With the exception of killifish, both the expression and protein sequences of the X-linked and Y-linked sex-determining genes vary; whereas in the other three species, only the expression of the Y-linked sex-determining gene is biased while the protein sequences of X-linked and Y-linked genes remain identical. In golden pompano and greater amberjack, the sex-determining gene *Hsd17b1* has also evolved through allelic divergence due to functional deficiency of Z-linked *Hsd17b1*. Regarding the biased expression of W-linked and Z-linked *Hsd17b1* as observed in the greater amberjack individuals (Koyama et al. 2019), this may be a subsequent outcome after emerging of the sex-determining gene and potentially caused by mutations located at the regulatory region. Interestingly, we did observe a minor peak approximately located approximately 219 kb upstream from the sex-determining mutation in the golden pompano (Fig. 2a). In comparison to distinct functions between the X-linked and Y-linked sex-determining genes in *O. dancena* (Takehana et al. 2014), *N. furzeri* (Reichwald et al. 2015), and *A. fimbria* (Herpin et al. 2021), the neutral evolution of Z-linked *Hsd17b1* may be a critical factor contributing to the long-term undifferentiated state of the sex chromosome.

## Conclusions

This study investigated the sex-determining gene and sex chromosomes in the golden pompano and the related species. Our findings demonstrate that the golden pompano and the greater amberjack, with the exception of sex-determining mutations, utilize the same sex-determining mechanism. We present a novel model to understand microevolution of the undifferentiated sex chromosomes and turnovers of sex-determining mutations. Our observations and simulation analysis provide evidence that the functional pattern of sex determining gene *Hsd17b1*, in conjunction with strong genetic drift and recombination, contribute to the turnover of sex-determining mutations and undifferentiated sex chromosomes.

## Materials and Methods

### Assembly of the Male Golden Pompano Genome

The genome of a male golden pompano was sequenced using both PacBio Sequel II and NovaSeq platforms, with subsequent genome assembly and annotation procedures detailed in supplementary materials.

### Assembly Validation with a Genetic Linkage Map

A full-sib family (F201803), consisting of two parents and 100 offspring, was subjected to resequencing in a previous study (Guo et al. 2020). The genetic linkage map was constructed using Lep-MAP3 (Rastas 2017) as previously described (Guo et al. 2019). The linkage groups were assigned with an LOD score of 14. The marker order within each linkage group was determined based on the best score from 10 independent runs.

### Calculation of the Time of Divergence

The divergence time between the golden pompano and the greater amberjack was estimated using MCMCTree in PAML version 4.9j (Yang 2007), with procedures detailed in supplementary materials.

### Investigation of the Sex Ratio in Families

A mass-cross population of the golden pompano (PM2018) was previously assigned to families using SSR markers (Guo et al. 2020). At 2-yr-old, 1,006 individuals were sampled, weighed, and their sexed determined through dissection gonad observation (supplementary table S1, Supplementary Material online). The number of females and males in each family was tallied, followed by a chi-square test to assess the hypothesis that their numbers are equal. An analysis of variance was conducted to evaluate the hypothesis that there is no disparity in body weight across sexes and families. The statistical analyses were performed using IBM SPSS statistics version 20 (IBM SPSS INC, Chicago).

### Improved Detection of Sex-Related Quantitative Trait Loci

Sex QTLs were identified through a combination of QTL mapping and GWAS analysis. The full-sib family (F201803) used to construct the genetic map was also included in the mass-cross population (PM2018). After removing 905 discordant SNPs between the genetic map and assembly, Lep-MAP3 (Rastas 2017) was utilized to reorder remaining SNPs. Sex QTLs were identified using MapQTL v6 (Ooijen et al. 2009). Potential QTLs were initially detected using the internal mapping model. Then, the SNP closest to the significant QTL was selected as a cofactor for subsequent mapping using the multiple QTL mapping model. In addition, sex identification and SNP calling were conducted for 82 natural individuals (37 males and 45 females) as previously described. The association between sex and genotype was assessed using a genotypic test in PLINK v1.90b6.2 (Purcell et al. 2007).

### The Characteristics of Sex Chromosomes

The read depth in the two parents of the full-sib family (F201803) was scanned in nonoverlapping 10-kb windows and compared as log2((male + 0.1)/(female + 0.1)). With the genetic map and physical map constructed, the local recombination rates of females were calculated using MareyMap v1.3 (Rezvoy et al. 2007) with the Loess-based method. The nucleotide diversity in the natural population was estimated in 10 kb nonoverlapping windows using VCFtools v0.1.16 (Koyama et al. 2019). The linkage disequilibrium in the natural population was estimated using LDBlockShow v1.36 (Dong et al. 2021).

### Validation of Alternative Splicing Events

RNA from the ovaries and testes of 1-yr-old individuals was subjected to long-read sequencing and RNA-seq, respectively. Two other sets of short reads from the ovary and testis were downloaded from the SRA; the individuals were of unknown age. For PacBio Iso-seq data processing, we used the Iso-seq3 pipeline (Gonzalez-Garay 2015) to construct FLNC transcripts. The transcript of *Hsd17b1* was identified by mapping the FLNC transcripts to the genome assembly using Minimap2 (Li 2018). The transcripts were constructed with StringTie v2.1.4 (Kovaka et al. 2019). The short reads from the Illumina platform were mapped to the genome assembly using HISAT2 (Kim et al. 2019) with a mapQ value above 30. Transcript expression was quantified with Ballgown (Pertea et al. 2016). Splicing events within the gene region of *Hsd17b1* were visualized as a Sashimi plot in Integrative Genomics Viewer (Thorvaldsdottir et al. 2013). The transcripts from the ovary and testis were also validated using Sanger sequencing.

### Sequence Alignment of *Hsd17b1*

The coding sequence of *Hsd17b1* were collected and the protein sequences were obtained with the ExPASy translate tool (Gasteiger et al. 2003). The sequences were aligned with MEGA X (Kumar et al. 2018) and ESPript v3.0 (Robert and Gouet 2014). The probability of rejecting the null hypothesis of neutral evolution was calculated by Z-test of codon-based test of neutrality. The phylogenetic tree was constructed as a neighbor-joining tree with *Hsd17b9* and *Hsd17b7* as outgroups. Robustness was tested with 1,000 bootstraps. The three-dimensional structure was predicted using SWISS-MODEL (Waterhouse et al. 2018) with human HSD17B1 (ID: 1iol.1) as a template, which was complexed with 17 beta-estradiol (Azzi et al. 1996).

### Simulations of the Microevolution of *Hsd17b1*

Based on observations in the golden pompano, the greater amberjack and zebrafish, we propose the hypothesis that the W-derived *Hsd17b1* were under purifying selection, Z-derived *Hsd17b1* evolved neutrally, and loss-of-function mutations leading to transformation from W chromosome to Z chromosome. We performed simulation analyses using a 1,678-bp segment of the *Hsd17b1* gene, comprising exonic and intronic regions, and the site Chr16:g.18219150A > G as the sex-determining mutation in the starting population. In each nonoverlapping generation, neutral point mutations were allocated at random positions at rate *μ* (1 × 10^−8^). Recombination occurred at a rate of *r* = *r*_0_ × (1% to 3% × *d*), in which *r*_0_ was set as the local recombination rate (1 × 10^−8^ or 1 × 10^−6^), *d* was the divergence between sequences, and the coefficient (3%) was selected according to the parameters in the neutral model (Jeffries et al. 2021). Missense mutations led to the transformation from W-derived segments to Z-derived segments, as well as base alterations at the alternative splicing sites. Population size (2*N*) was limited to 100 or 10,000. Simulations were run for 100,000 generations, and 100 repetitions were performed for four sets of parameters (Table 1). The average number of nucleotide differences between segments in the whole population (*π*_all_), the Z-derived segments (*π*_z_), the W-derived segments (*π*_w_), and the average number of nucleotide differences between W-derived segments and Z-derived segments (*d_xy_*) were calculated, as well as the fixation indices (Fst).

### The Detection of Sex-Determining Mutations in Florida Pompano

Seven males and 14 females of Florida pompano, were utilized to examine any changes in the focal SNPs. Phenotypic sex, re-sequencing and read mapping were conducted as previously described. The association analysis was conducted with PLINK v1.90b6.2 (Purcell et al. 2007). The sex-determining mutation were manually checked using SAMtools v1.9's text alignment viewer (Li et al. 2009).

### Generation of Zebrafish Knockout Lines

AB-strain zebrafish obtained from the China Zebrafish Resource Center (CZRC, Wuhan, China) were utilized for gene knockout. CRISPR/Cas9 target sites were designed using ZiFiT Targeter online software (Sander et al. 2010), which identified the sequence 5′GG-(N18)-NGG3′ in the first exon (CCTTGCTGTGCATCTCGCATCAA), the second exon (CTATGCTACTATGCGGAACTTGG), and the third exon (AGGACCCATAAGACCCACAC and GGACACTATAAGAGCCATCC) of *Hsd17b1* in zebrafish (ID: ZDB-GENE-040901-5, ZFIN). Each sgRNA was synthesized through the cloning of annealed oligonucleotides into the sgRNA expression vector pT7-gRNA, followed by in vitro transcription. The pSP6-2sNLS-spCas9 plasmid was linearized by XbaI, and capped Cas9 mRNA was synthesized using a T7mMESSAGE Ultra Kit (Ambion). Concentrations of capped mRNAs were measured with a NanoDrop instrument (Thermo Scientific, Waltham, MA), and their quality was examined through agarose gel electrophoresis. Microinjection was performed on zebrafish embryos at the one-cell stage, with a co-injection of 100 pg sgRNA and 600 pg Cas9 RNA were. Noninjected embryos were used as controls. All embryos were maintained in an environmental incubator at 28 °C for at least 1 d prior to viability examination. The male and female F1 fish exhibiting identical frameshift mutations were crossed to generate homozygous F2 mutants (−/−). Mutations were confirmed via directed Sanger sequencing.

### Assessment of the Impact of *Hsd17b1* Mutation in Zebrafish

Fish were sexed based on secondary characteristics (Romano et al. 2020) and confirmed with hematoxylin–eosin staining of gonads on day 145 postfertilization. Male fertility of the homozygous F2 mutants was evaluated by mating performance, defined as the ability to induce spawning of wild-type females. At least two male fish of each genotype (+/+ or −/−) were tested with wild-type (+/+) female fish separately, with tests repeated at 5-d intervals. The spawning rate was calculated as the proportion of successful spawning pairs to the total number of pair after 24 h. The experiment was repeated no less than three times.

## Supplementary Material

Supplementary material is available at *Genome Biology and Evolution* online

## Supplementary Material

evae037_Supplementary_Data

## Data Availability

Genome assembly: GCA_022709315.1. Genome sequencing: SRR12380975 (PacBio genome sequencing), SRR12380974 (Illumina genome sequencing), SRR12380973 (PacBio transcriptome sequencing), and SRR8168440 (Hi-C sequencing). Iso-seq: SRR14553106 (female), SRR14553104 (male). RNA-seq: SRR6168954 (female), SRR14553107 (female), SRR14553103 (female), SRR6168955 (male), SRR14553105 (male), SRR14553102 (male). Resequencing: PRJNA552381 (full-sib family of the golden pompano, F201803, *n* = 102), PRJNA730209 (golden pompano, *n* = 82), PRJNA917782 (Florida pompano, *n* = 21). W-derived *Hsd17b1* coding sequence: OK170040, Z-derived *Hsd17b1* coding sequencing: OK170041. The code for simulation of turnover of sex-determining mutations is available at https://github.com/zsdxgl/Turnover-of-sex-mutation.
