# Supplementary material for: Turnovers of Sex-Determining Mutation in the Golden Pompano and Related Species Provide Insights into Microevolution of Undifferentiated Sex Chromosome
Source: Genome Biol Evol. 2024 Feb 26;16(3):evae037. doi: 10.1093/gbe/evae037 (PMC10919887; doi:10.1093/gbe/evae037)
Supplement: evae037_Supplementary_Data [file evae037_supplementary_data.docx]

Turnovers of sex-determining mutation in the golden pompano and related species provide insights into microevolution of undifferentiated sex chromosome

Liang Guo^1,2^*, Danilo Malara^3^, Pietro Battaglia^4^, Khor Waiho^5,6,7^, D. Allen Davis^8^, Yu Deng^1^, Zhongyuan Shen^1^, and Ke Rao^1^

^1^State Key Laboratory of Developmental Biology of Freshwater Fish, Hunan Normal University, Changsha, Hunan Province, China, 410081

^2^South China Sea Fisheries Research Institute, Chinese Academy of Fishery Sciences, Guangzhou, Guangdong Province, China, 510300

^3^Stazione Zoologica Anton Dohrn, Integrated Marine Ecology Department, CRIMAC, Calabria Marine Centre, Amendolara, Italy, 87071

^4^Integrated Marine Ecology Department, Stazione Zoologica Anton Dohrn, Sicily Marine Centre, Messina, Italy, 98168

^5^Higher Institution Centre of Excellence (HICoE), Institute of Tropical Aquaculture and Fisheries, Universiti Malaysia Terengganu, Kuala Nerus, Terengganu, Malaysia, 21300

^6^Centre for Chemical Biology, Universiti Sains Malaysia, Minden, Malaysia, 11900

^7^Department of Aquaculture, Faculty of Fisheries, Kasetsart University, Bangkok, Thailand, 10900

^8^School of Fisheries, Aquaculture and Aquatic Sciences, Auburn University, Auburn, Alabama, 36830

*Correspondence: Liang Guo, State Key Laboratory of Developmental Biology of Freshwater Fish, Hunan Normal University, Changsha, Hunan Province, China, 410081

**Supplementary Materials and Methods**

1. Assessing genome assembly thorough family-based genome wide association study

We have assessed the previous genome assembly of golden pompano (GCA_900231065.1) using Hi-C read in a prior study and identified obvious contig misplacement(Guo *et al*. 2021). To further evaluate the sex chromosome assembly, we conducted a family-based genome wide association study.

We have collected a full-sib family (F201803) consisting of 2 parents and 100 offspring, which was submitted to resequencing in our previous research (Guo *et al*. 2020). The raw reads were filtered using Trimmomatic v0.38 (BolgerLohse and Usadel 2014) with default parameters, followed by mapping to the reference genome assembly (GCA_900231065.1) using BWA-MEM v0.7.17 (Li 2013) and manipulation of SAM/BAM format files using SAMtools v1.9 (Li *et al*. 2009). Only accurately mapped reads were selected for genotyping. The MarkDuplicates tool in GATK v4.1.1.0 (McKenna *et al*. 2010) was used to identify and remove duplicated reads, while the Haplotypecaller tool was employed to perform hard filtering of SNPs based on the quality metrics (QUAL<5,000, depth>3,000, quality by depth<2, phred-scaled Fisher's exact test p-value>60, root mean square mapping quality<30, mapping quality Mann–Whitney rank-sum<−10, mapping quality Mann–Whitney rank-sum>10, read position Mann–Whitney rank-sum<−10, read position Mann–Whitney rank-sum>10, strand odds ratio>3). A genome wide association study (GWAS) was performed using genotypic test using with v1.9(Purcell *et al*. 2007). The *p-*values were displayed using the R package qqman (Turner 2014). The Manhattan plot is presented in Fig. S1, revealing five peaks that suggest at least five segments from sex chromosome were misplaced.

1. genome assembly construction for golden pompano

High quality DNA was isolated from muscle of a male golden pompano. A 300-bp paired-end Illumina fragment library was constructed and sequenced on the NovaSeq platform (Illumina, CA, USA). 301.05 M paired-end reads (141×) were produced and utilized to estimate genome size and polish the genome assembly. A 40-kb library was constructed and sequenced on the PacBio Sequel II platform (Pacific Biosciences, CA, USA). PacBio sequencing produced 6.72 M subreads (>500 bp). From each polymerase read, the median-length subread was extracted, and 5.87 median-length subreads (142×) were used for genome assembly. The average and N50 lengths of these median-length subreads were 16.12 kb and 26.90 kb, respectively. RNA was extracted from the gill, brain, heart, liver, spleen, kidney, stomach, intestine, skin, fin, muscle, testis and ovary. Equal amounts of RNA from each tissue were mixed to generate one sample. An Iso-seq library was prepared and sequenced on two cells of a PacBio Sequel system (PacBio, CA, USA). A total of 153 K full-length and non-chimeric consensus (FLNC) sequences were produced using SMRT Link 5.0 software. In addition, 402.29 M paired-end reads from RNA-seq of eight tissues, namely, muscle, liver, spleen, kidney, stomach, skin, brain, gill, gonad and intestine, and 382.80 M paired-end reads from Hi-C data were obtained from a previous study(Zhang *et al*. 2019).

Genome size and heterozygosity were estimated using Jellyfish v2.2.10 (Marçais and Kingsford 2011) and GenomeScope v1.0 (Vurture *et al*. 2017) with 84 M Illumina genome reads (~40×). PacBio median-length subreads were subjected to assembly with the Falcon toolkit v1.8.1(Chin *et al*. 2016). The primary contigs were polished with PacBio long reads using Arrow v2.3.3 (Chin *et al*. 2013) and Illumina short reads using Pilon v1.23 (Walker *et al*. 2014). The polished contigs were retrieved with nucleotide sequences from NCBI-nr database with blastn (Altschul *et al*. 1997) to remove contaminants. A total of 382 M Hi-C paired-end read pairs were used to anchor the contigs into scaffolds. Approximately 43.30% of reads were mapped to the contigs with Juicer(Durand *et al*. 2016). The chromosome-level assembly was obtained with a 3D-DNA pipeline(Matthews *et al*. 2018). The assembly was manually curated with Juicebox v1.11.01(Durand *et al*. 2016). The gaps in the pseudochromosomes were filled with PBJelly v15.8.24 (English *et al*. 2012). Then, the gap-filled scaffolds were polished again as described above.

The completeness of the final genome assembly was assessed using BUSCO v4.0.5 with actinopterygii_odb10 (Sim O *et al*. 2015). The quality of the final assembly was also assessed with the alignment rate. The median-length PacBio genome reads were mapped with minimap2 v2.17 (Li 2018). The FLNC sequences were mapped with GMAP v2020-06-30 (Wu and Watanabe 2005). RNA-Seq reads were mapped with HISAT2 v2.1.0 (Kim *et al*. 2019).

1. Annotation of the genome assembly

Transposable elements were detected and classified using RepeatMasker v4.0.9 and the Dfam_3.0 database (Hubley *et al*. 2016), in which the query species group was set to Teleostei. Gene structure was predicted using MAKER v2.31.10 (Cantarel *et al*. 2008). The first-round annotation was run to predict gene models from transcript evidence and protein evidence with an ab initio predictor, Augustus v3.3.3 (Stanke *et al*. 2006). The transcript evidence included FLNC sequences and the RNA-seq transcript assembly. RNA-seq reads were mapped to the final genome assembly with HISAT2 v2.1.0 (KimLangmead and Salzberg 2015), and the transcript assembly was predicted with StringTie v2.0.3 (Pertea *et al*. 2016) by using the conservative model (--conservative). The protein evidence included the proteins from the uniprot_sprot database (Apweiler *et al*. 2004). The program Augustus was trained with conserved genes in BUSCO v4.0.5. The second round annotation was run to predict gene models from transcript evidence and protein evidence with ab initio predictors, namely, SNAP v2006-07-28 (Korf 2004) and Augustus v3.3.3 (Stanke *et al*. 2006). SNAP was trained with complete gene models surrounded by genomic sequences obtained in the first-round annotation with AED set to 0.5. Augustus was trained with the predicted proteins in the first-round annotation. The descriptive statistics of gene models were obtained for the primary transcripts by Eval v2.2.8(Keibler and Brent 2003). Functional annotation was performed with eggNOG-mapper v2 (Huerta-Cepas *et al*. 2017), which was based on orthology assignments. Noncoding RNAs, including rRNAs, snRNAs, miRNAs, and tRNAs, were identified using Infernal v1.1.2 (Kolbe and Eddy 2009) with the Rfam database 14.1 (Kalvari *et al*. 2018).

Annotated transposable elements accounted for only 4.17% of the assembly (Table S9). Gene prediction identified 25,720 protein-coding genes (Table S10), with an average length of 10669.7 bp for the full gene sequence, 1623.8 bp for the coding sequence, 169.8 bp for the exon, and 924.9 bp for the intron. These values are comparable to those of 14 well-annotated fish species (Table S11-12). A total of 24,177 genes (94.0%) were annotated with putative functions, with 64.6% and 67.9% assigned gene ontology and KEGG orthology terms, respectively (Table S13). Noncoding RNA prediction yielded 1,155 tRNAs, 431 rRNAs, 188 snoRNAs, 356 snRNAs, 4 lncRNAs, 961 microRNAs, and 407 cis-regulatory elements, with a total length of 369.6 kb (Table S14).

1. Calculation of divergence time

In addition to the sequences of the golden pompano, the protein sequences of 14 other fish species were downloaded from the Ensembl database (Flicek *et al*. 2013) and included in phylogenetic analysis. Primary coding sequences and protein sequences of each gene were extracted, while those containing termination codons or unknown bases were excluded. Gene families were identified using OrthoFinder2 (Emms and Kelly 2019). The protein sequences of the single-copy genes were aligned using MAFFT v7.450 in auto mode (Kuraku *et al*. 2013), and corresponding nucleotide sequence alignment was obtained with PAL2NAL v14 (SuyamaTorrents and Bork 2006). A phylogenetic tree was constructed using IQ-TREE v1.6.2 (Nguyen *et al*. 2015) with extended model selection and 1000 ultrafast bootstraps, where the GTR+F+R3 model was selected based on Bayesian information criterion. The divergence time was estimated using MCMCTree in PAML version 4.9j (Yang 2007). Two calibration time points based on fossil records of takifugu-tetraodon (32.25~56.0 Ma) and zebrafish-medaka (149.85~165.2 Ma)(Benton and Donoghue 2007; Yang *et al*. 2016) were integrated, and the age of the root was constrained as the origin of modern ray-finned fishes (approximately 385 Ma)(Giles *et al*. 2017). The prior for substitutions per site per year was set at 10^-8^. The independent rate model was employed, and convergence was validated by comparing the posterior mean times across repeated analyses (Dos Reis and Yang 2019). The time tree was visualized using MCMCTreeR (Puttick 2019).

We obtained primary protein sequences of 14 fish species from Ensembl database (Table S15) and identified 3,093 single-copy orthologous genes through orthology assignment (Table S16-19). Phylogenetic trees were constructed using all the genes and with fourfold degenerate synonymous sites from single-copy genes (352,840 bp), respectively. Both trees were identical, and all nodes were supported with 100% bootstraps. Our analysis revealed that the golden pompano and the greater amberjack diverged approximately 57.45 million years ago (95% confidence interval: 40.82~74.32 million years ago) based on the independent-rate model (Fig S4). This result was consistent with the topological structure and divergence times of other studies including common species *Danio rerio*, *Oryzias latipes*, *Gasterosteus aculeatus*, *Takifugu rubripes*, and *Tetraodon nigroviridis* (Yang *et al*. 2016).


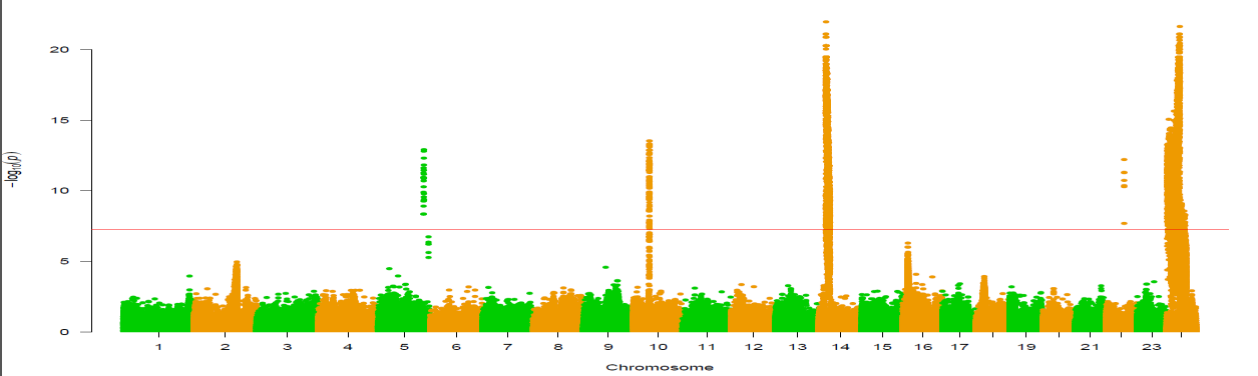


**Fig S1**. The Manhattan plot of a family-based GWAS using the previous genome assembly as reference (GCA_900231065.1). According to survey of sex ratio and QTL-mapping, the sex is determined by a single genetic factor in the golden pompano. However, five peaks were detected in this study, indicating the sex chromosome were misassembled and assigned into at least five scaffolds.





**Fig S2**. The scatter diagram of the depth of coverage and GC content of the genome assembly (GCA_022709315.1). The depth of coverage was assessed by mapping the resequencing reads to the genome assembly. The GC content was scanned in non-overlapped windows of 10 kb.


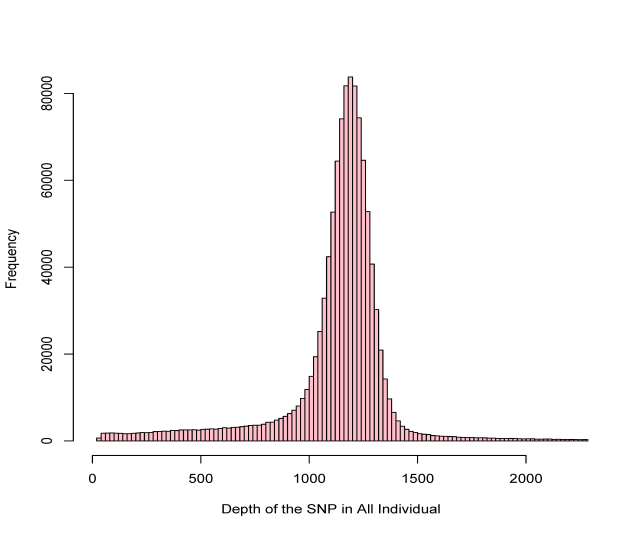

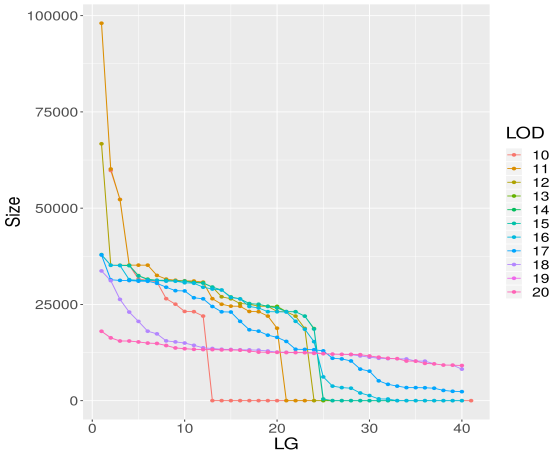


b

a

**Fig S3**. The construction of genetic linkage map for the golden pompano. a. The coverage depth of the selected SNPs with quality score of 999 in all the individuals from the full-sib family (F201803). b. The change of linkage group number with various LOD thresholds. In the software Lep-MAP3 (Rastas 2017), two parameters have to be set, the cutoff threshold of SNP number in each linage group and the best LOD. When the best LOD chosen, the SNPs are expected to be assigned into stable number of linkage group. To select the best LOD, the parameter of marker number in each linkage group were set as infinity and the stable number of linkage group is expected. The linkage group number is 24 when LOD set to 13-15.


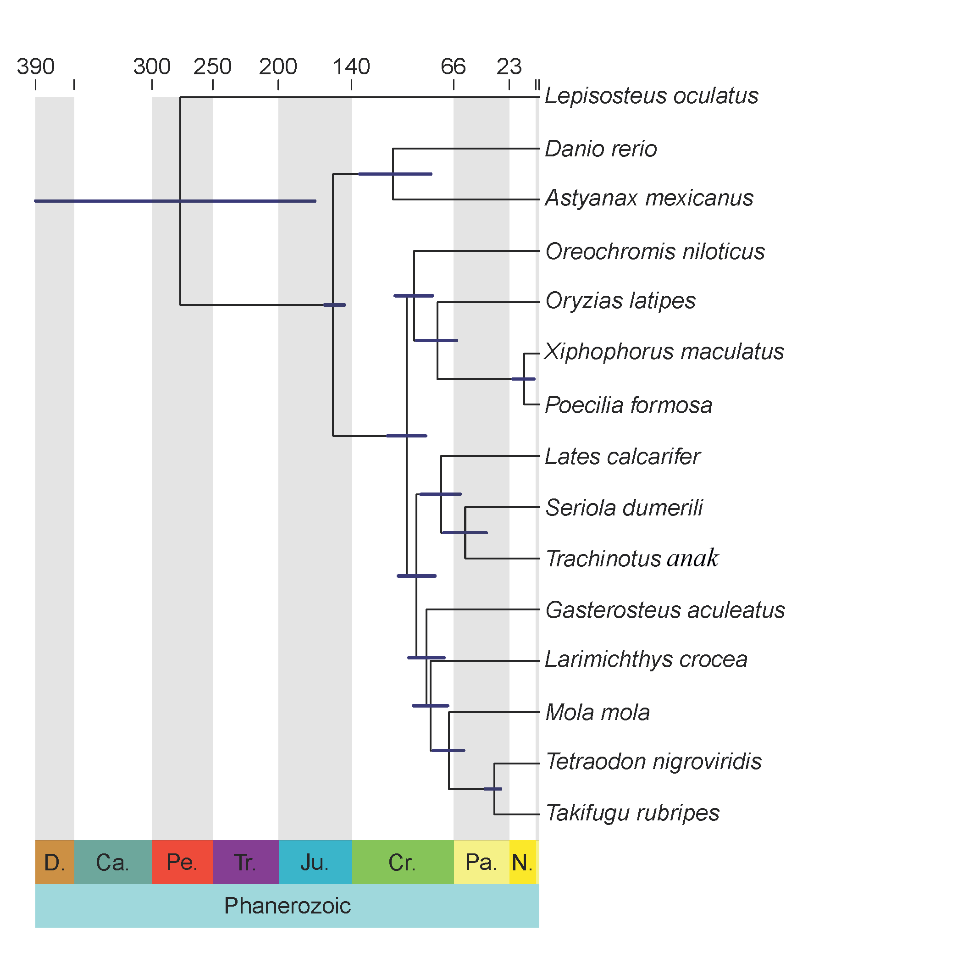


a

b

**Fig S4**. The divergence between the golden pompano and the greater amberjack. a. The golden pompano and the greater amberjack diverged at 57.45 million years ago. b. the comparison of posterior mean times between two replicaed estimations. The arrows indicating the two calibration time points (takifugu-tetraodon: 32.25~56.0 Ma; zebrafish-medaka: 149.85~165.2 Ma) (Benton and Donoghue 2007; Yang *et al*. 2016)


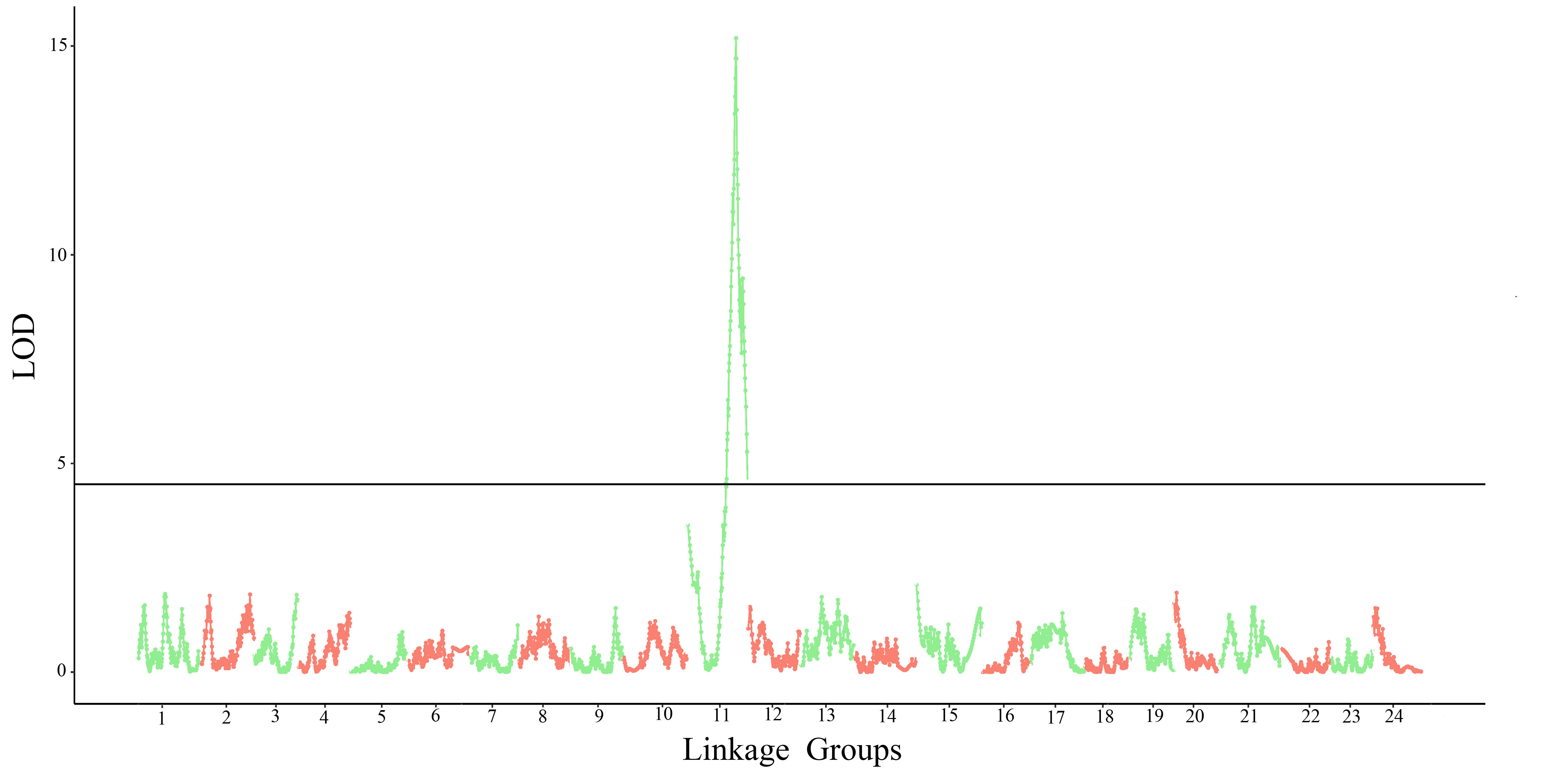


a


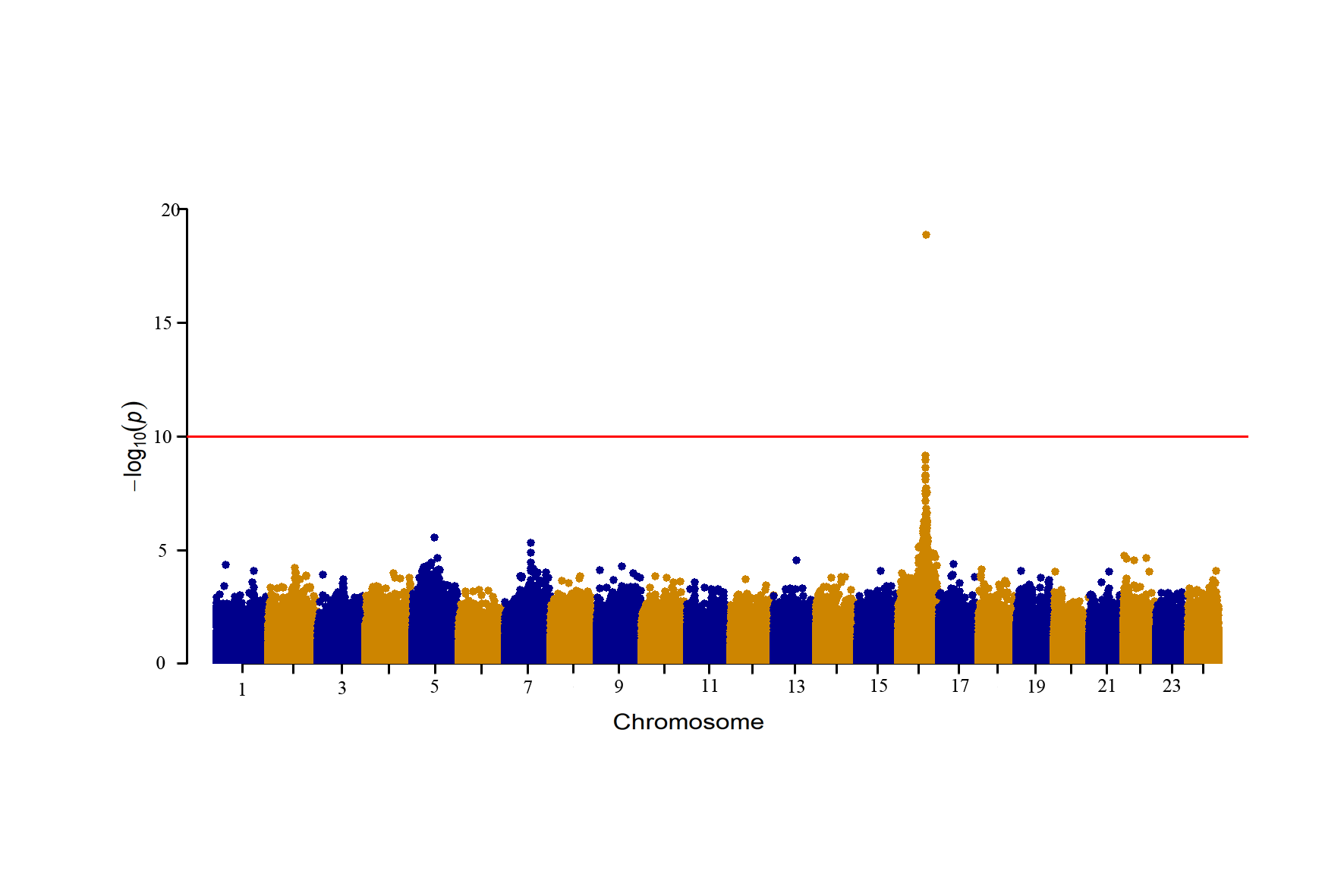


b
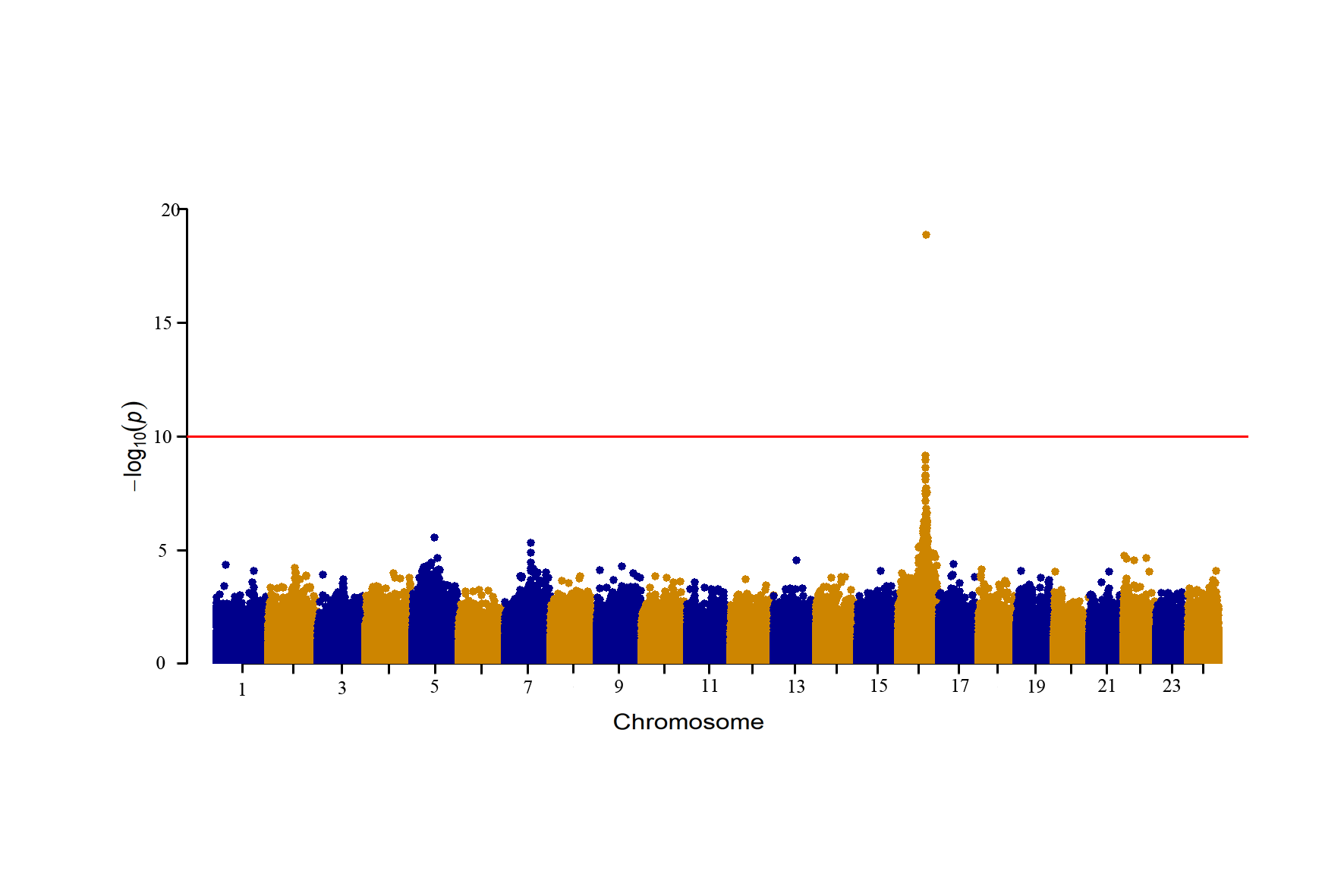


**Fig S5**. Detection of sex QTL in the golden pompano*.* a. The QTL mapping of the sex; b. GWAS analysis of the sex. The linkage group 11 in the genetic linkage map is corresponding to the Chr16 in the genome assembly.


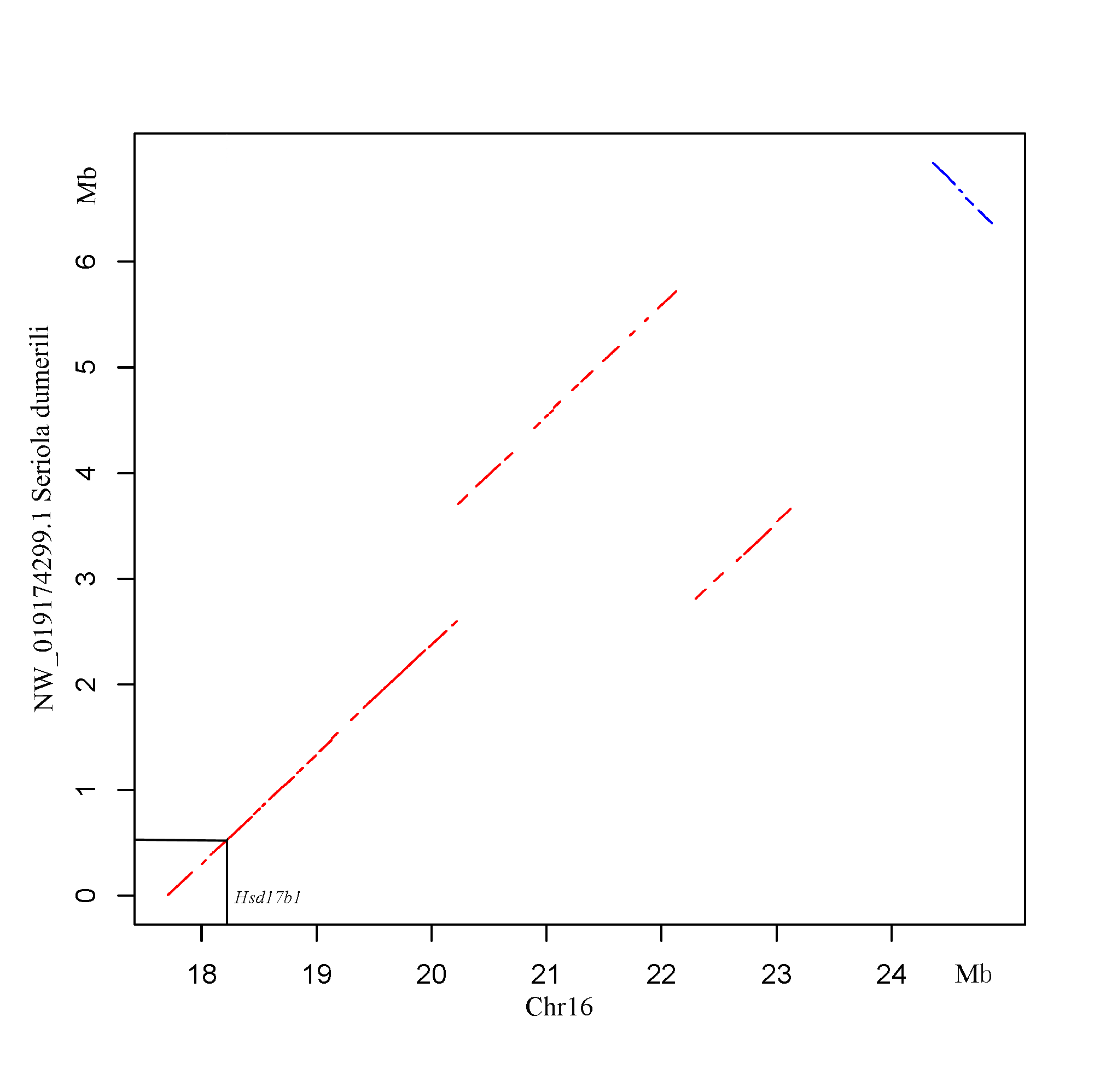


**Fig S6**. The alignment between the scaffold containing *Hsd17b1* in the greater amberjack and Chr16 of the golden pompano.


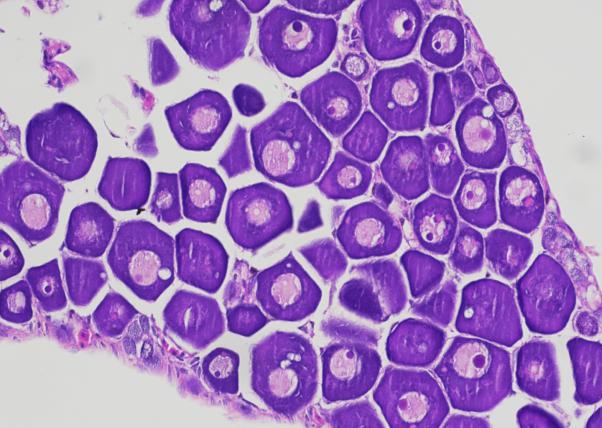

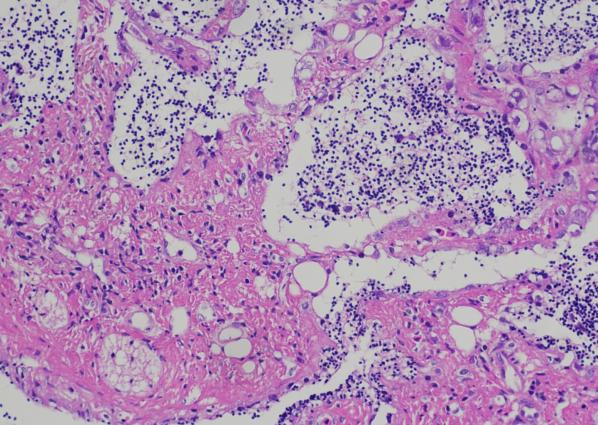


a


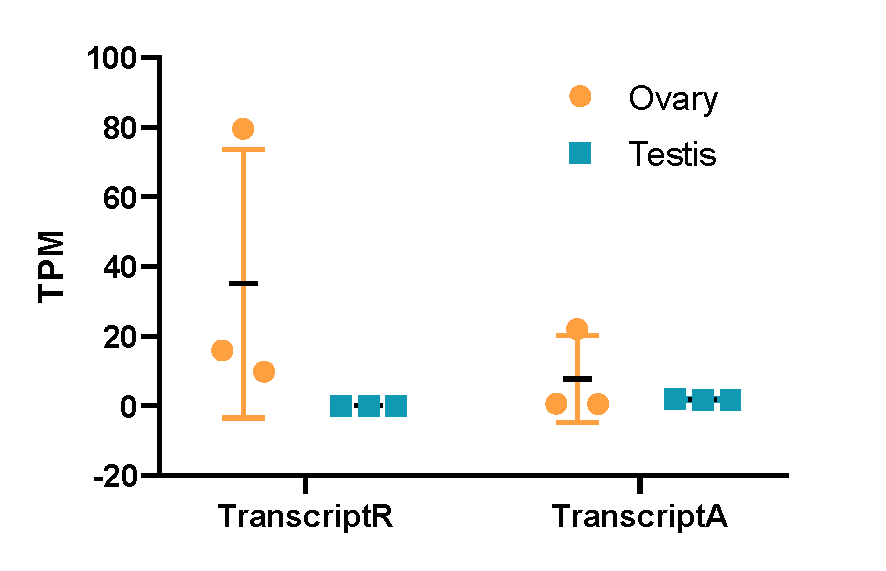


b


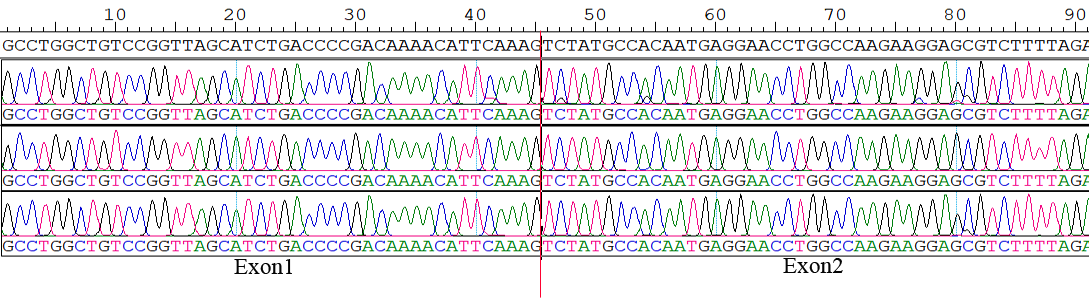

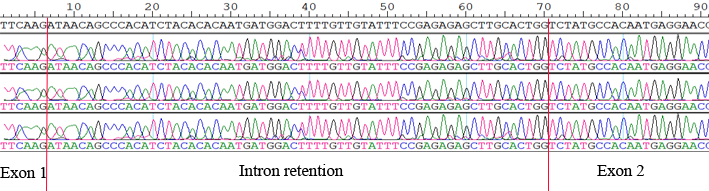


d

c

**Fig S7**. The expression of *Hsd17b1* in the golden pompano. a. The histologic section of one-year ovary (left, 400×) and testis (right, 400×). b. The TPM (Transcripts Per Kilobase of exon model per Million mapped reads) of the Z-derived transcript (TranscriptA) and W-derived transcript (TranscriptR) of *Hsd17b1* in gonads. c. Sanger sequencing of the *Hsd17b1* mRNA from ovaries. d. Sanger sequencing of the *Hsd17b1* mRNA from testes.


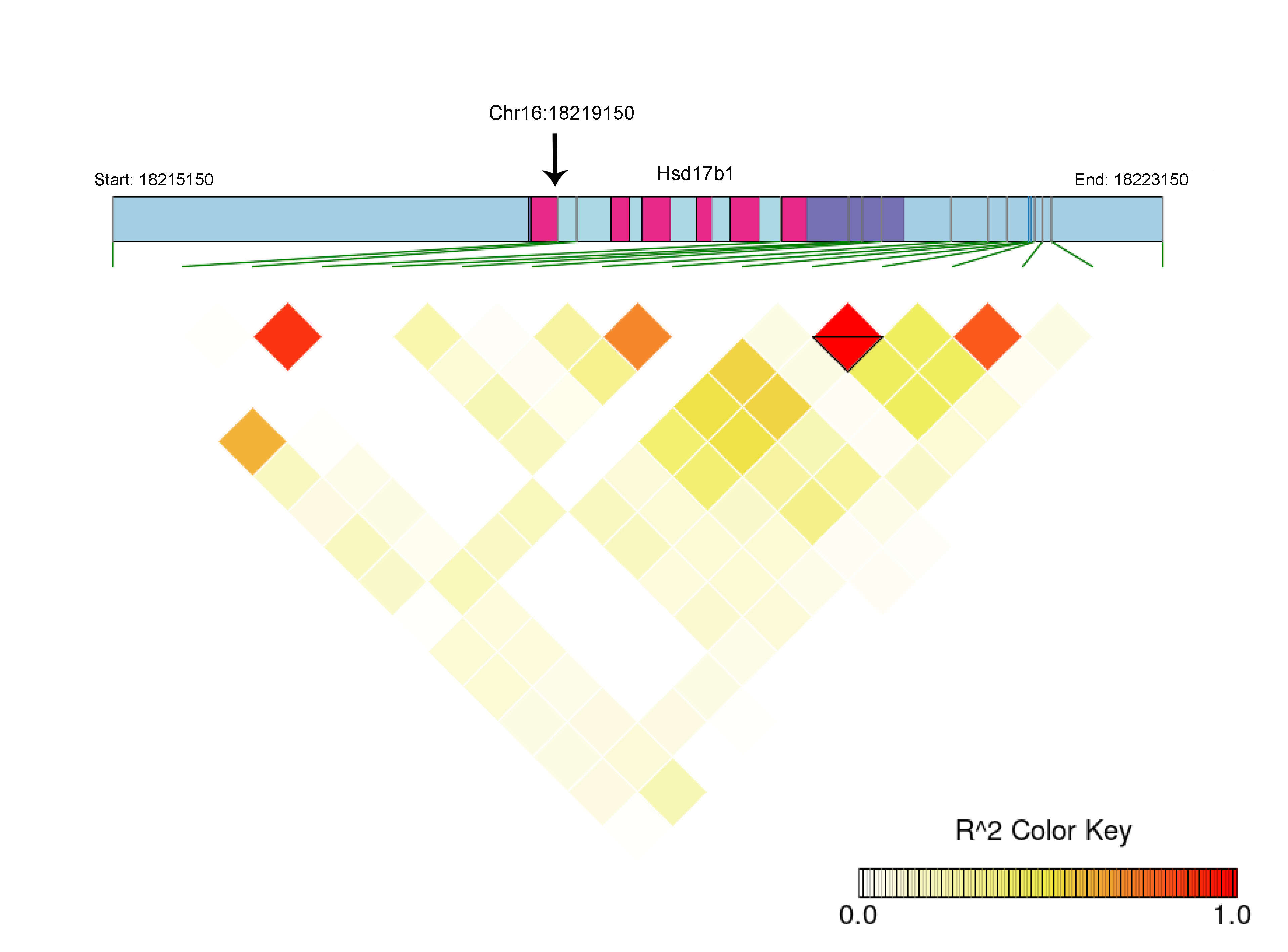


**Fig S8**. Linkage disequilibrium of the SNPs flanking of the sex determining SNP (Chr16:g.18219150A>G) in the natural population of the golden pompano.





**Fig S9**. The estimation of the recombination rate in the proto-sex chromosome using the female individuals in the natural population of the golden pompano.


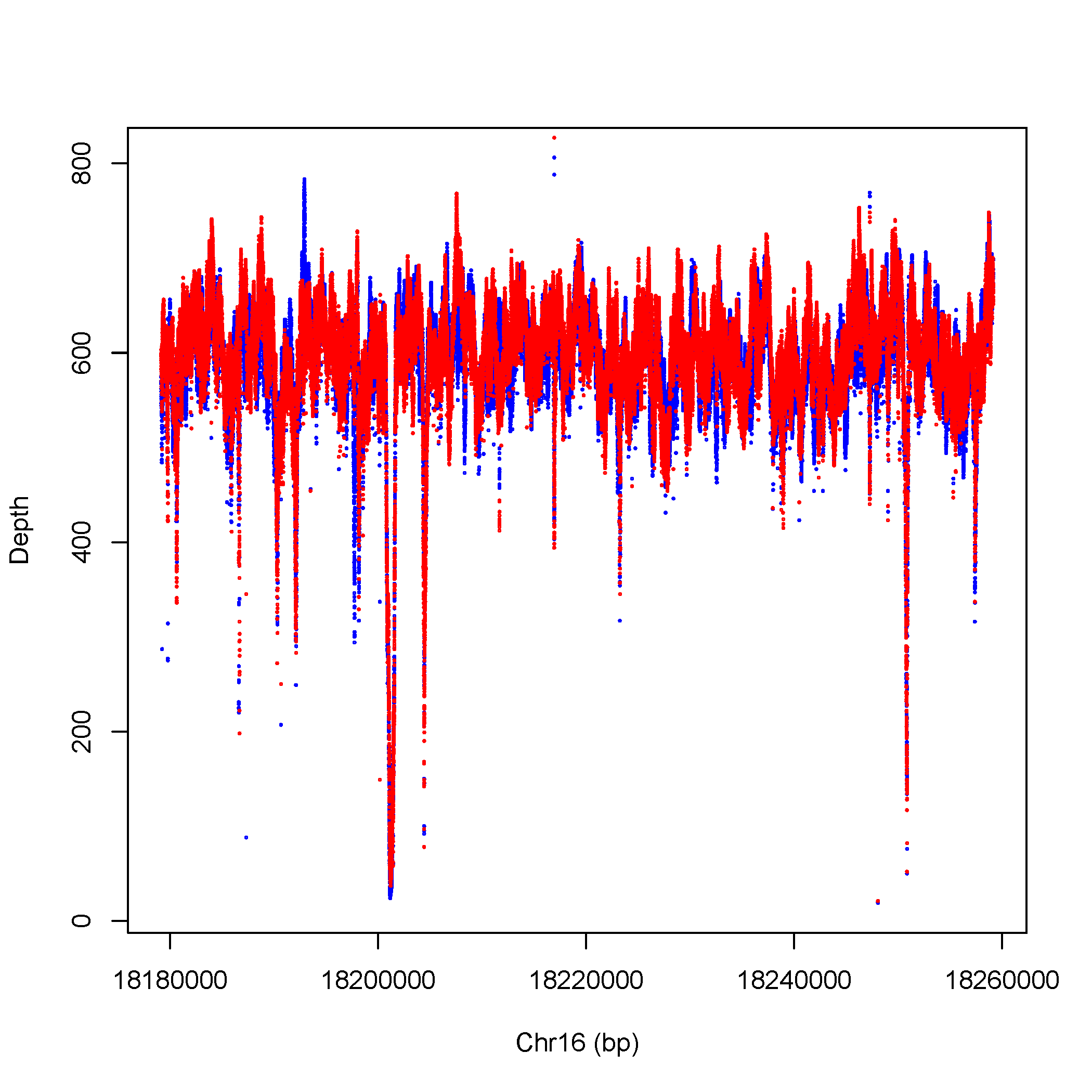


**Fig S10**. The depth comparison between females and males from the full-sib family of the golden pompano. Blue points represent 50 female progeny and the female parent, and red points represent 50 male progeny and the male parent in the full-sib family.


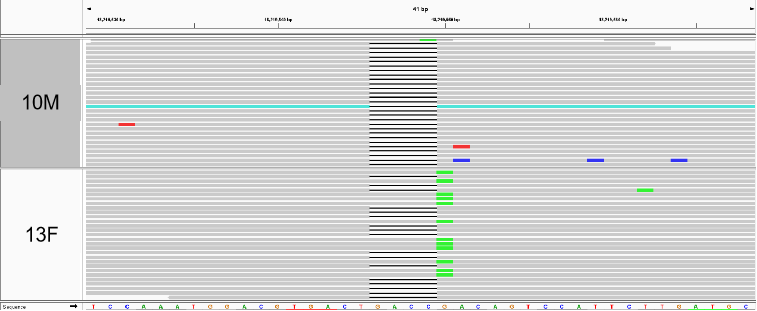


**Fig S11.** The genotypes of the sex-determining mutation in Florida pompano. The figure shows the genotypes of two samples, 10M and 13F, which are male and female, respectively. The sex-determining mutation is a 4-bp deletion in the exon2 of *Hsd17b1* from Chr16:18219546 to Chr16:18219549. The genotypes in female and male are GACC/- and -/-. The reference is the genome assembly of the golden pompano (GCA_022709315.1).


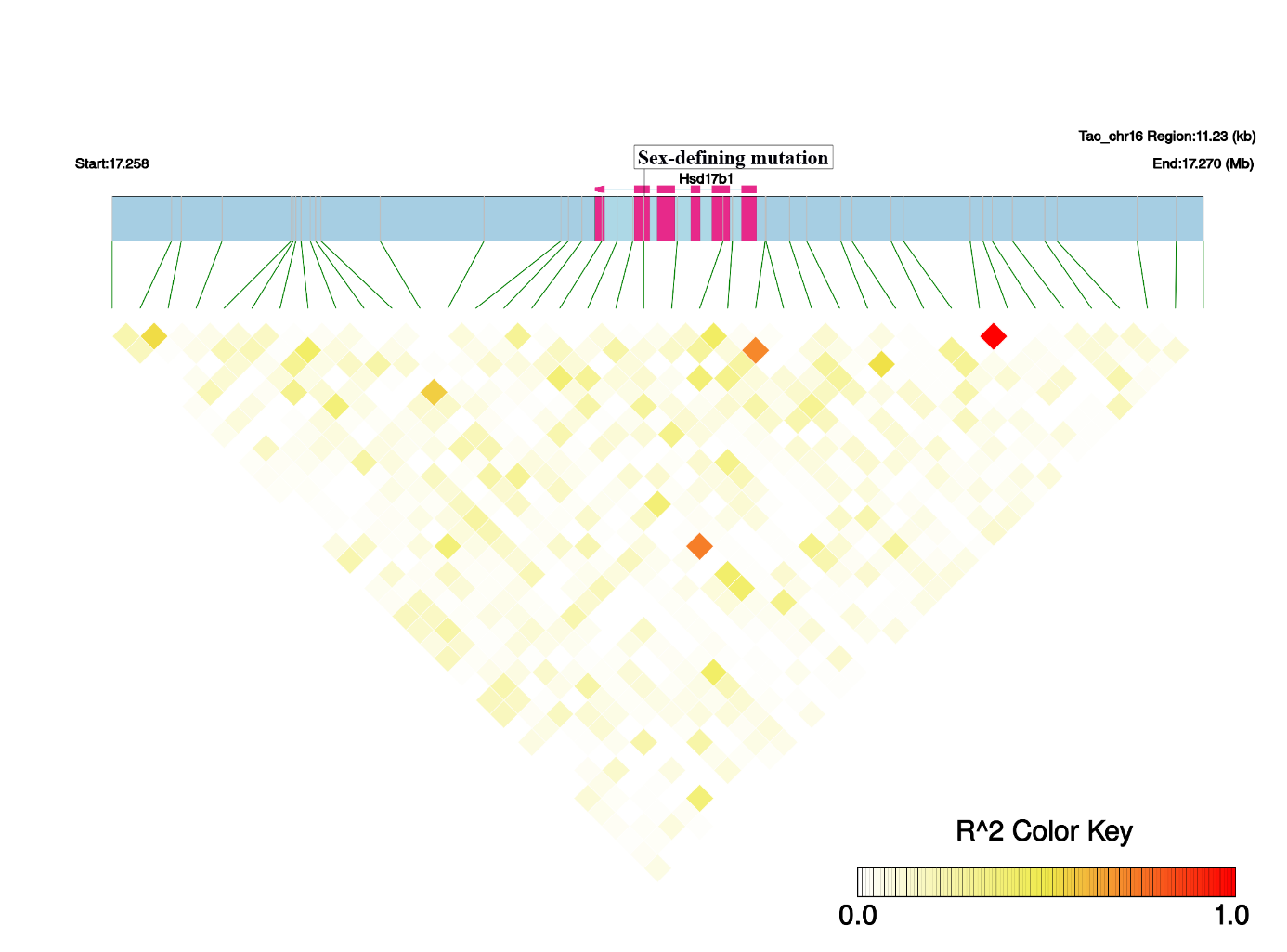


Fig S12. The linkage disequilibrium around the sex determining mutation in Florida pompano population. The linkage disequilibrium is measured by r^2^.


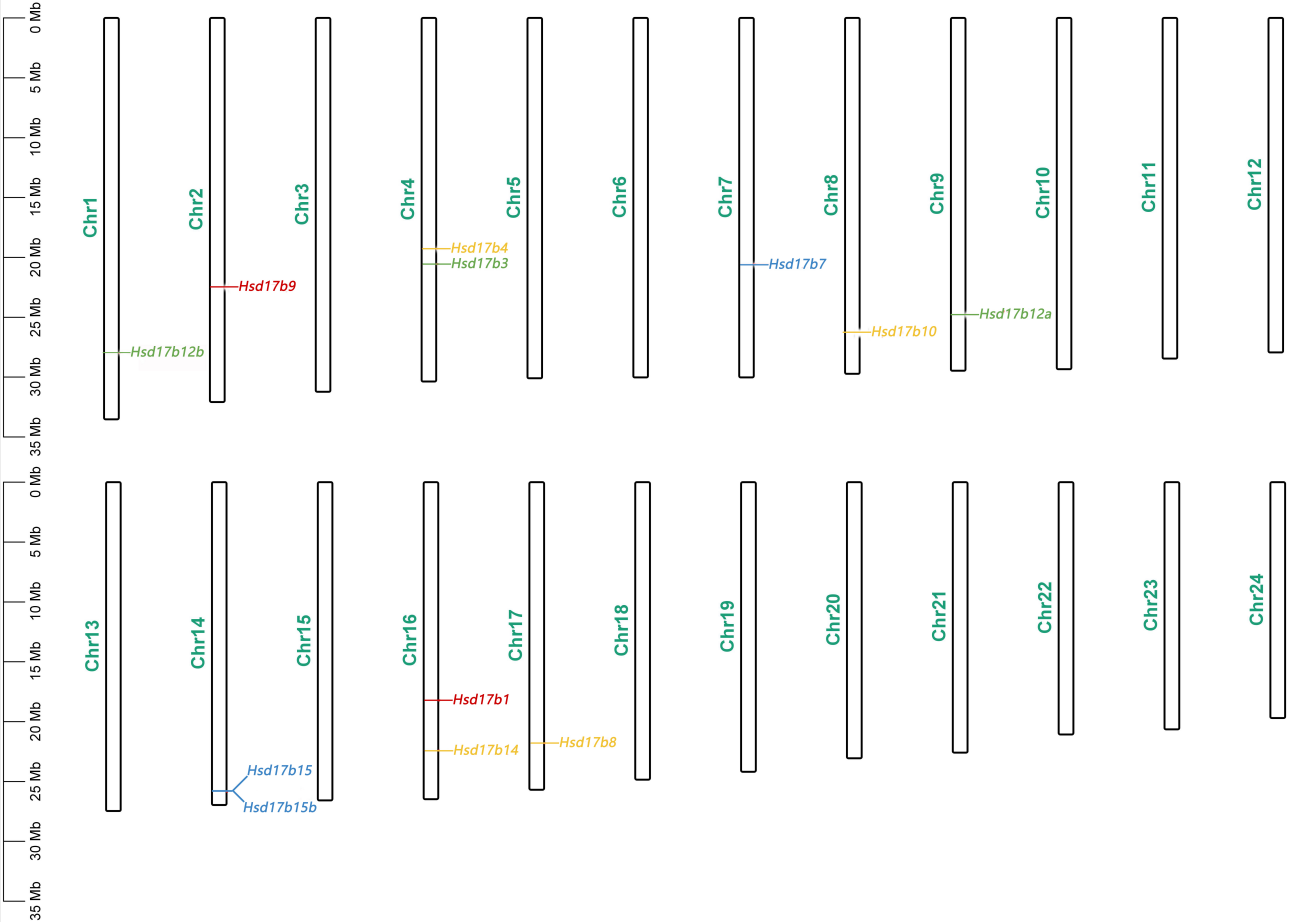


**Fig S13**. The gene family of 17β-hydroxysteroid dehydrogenases in the golden pompano. The gene sequences of *Hsd17b1* (XM_020094445.1), *Hsd17b3* (XM_020102701.1), *Hsd17b4* (XM_020099319.1), *Hsd17b7* (XM_020080974.1), *Hsd17b8* (XM_020107600.1), *Hsd17b9* (XM_020096611.1), *Hsd17b10* (XM_020111967.1), *Hsd17b12a* (XM_020079397.1), *Hsd17b12b* (XM_020099439.1), *Hsd17b14* (XM_020094542.1), *Hsd17b15* (XM_020107568.1) from *Paralichthys olivaceus* (Zou *et al*. 2020) were used to search the genome assembly of the golden pompano with blastn. The alignments were also manually checked. There is only one *Hsd17b1* detected.


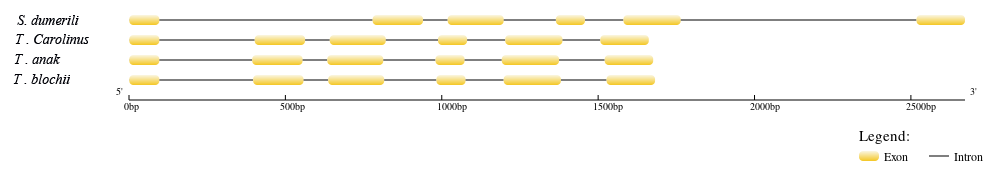


**Fig S14**. The gene structure of W-linked *Hsd17b1* in the four species*.* The figure was generated using GSDS 2.0 (Gao *et al*. 2015)*.* The sequence differences were mainly accumulated in the first and fifth introns between the greater amberjack and other three species.

**Table S1**. The statistics of sex records in a mass-cross population (PM2018). Five full-sib families were identified using SSR markers.

|  | Individual Number | | | Weight (g, mean±SD) | | |
| --- | --- | --- | --- | --- | --- | --- |
|  | Female | Male | All | Female | Male | All |
| Family1 (F201803) | 169 | 192 | 361 | 964.02±173.85 | 905.56±129.54 | 932.93±154.47 |
| Family2 | 183 | 184 | 367 | 967.57±159.31 | 880.44±155.99 | 923.88±163.37 |
| Family3 | 112 | 91 | 203 | 1013.97±158.67 | 926.39±135.16 | 974.71±154.53 |
| Family4 | 19 | 18 | 37 | 1002.05±184.27 | 908.95±107.32 | 956.76±156.98 |
| Family5 | 19 | 19 | 38 | 874.57±266.62 | 782.16±224.91 | 828.36±247.75 |
| All | 502 | 504 | 1006 | 974.51±171.80 | 895.62±146.55 | 934.99±164.38 |

**Table S2.** The statistics for genome sequencing for the golden pompano*.*

|  | Tissue | Sequencing | Read/Read Pair (M) | Base (Gb) | Coverage | Alignment Ratio |
| --- | --- | --- | --- | --- | --- | --- |
| DNA | muscle | Hi-C | 382.80 | 114.84 | 179.65 | 43.30%^a^ |
|  | muscle | PacBio | 5.87 | 94.66 | 148.08 | 86.09%^b^ |
|  | muscle | PE150 | 301.05 | 90.31 | 141.28 | 99.57% |
| RNA | mixed | PacBio | 0.26 | 4.42 |  | 98.70%^c^ |
|  | ovary | PE150 | 19.92 | 5.98 |  | 92.10% |
|  | blood | PE150 | 21.51 | 6.45 |  | 87.19% |
|  | brain | PE150 | 20.08 | 6.02 |  | 84.38% |
|  | intestines | PE150 | 741.16 | 22.23 |  | 93.56% |
|  | lever | PE150 | 90.54 | 27.16 |  | 95.90% |
|  | muscle | PE150 | 46.90 | 14.07 |  | 96.49% |
|  | kidney | PE150 | 5.51 | 1.65 |  | 81.33% |
|  | spleen | PE150 | 20.00 | 6.00 |  | 94.43% |
|  | fin | PE150 | 24.01 | 7.20 |  | 94.68% |
|  | gill | PE150 | 39.07 | 11.72 |  | 92.76% |
|  | stomach | PE150 | 23.32 | 7.00 |  | 90.93% |
|  | testis | PE150 | 17.27 | 5.18 |  | 90.27% |

^a^ Hi-C contacts with a mapQ score ≥ 30.

^b^ The mapped median-length subread.

^c^ The mapped FLCN sequences.

**Table S3**. The general statistic of the genome assembly of the golden pompano.

| Items | | Number |
| --- | --- | --- |
| Raw Reads | Total Base | 107085406247 |
|  | Coverage | 152.979 |
|  | Mean Length | 16357.8 |
|  | N50 | 26156 |
|  | Read Number | 6546443 |
| Median Length Reads | Total Base | 94615201124 |
|  | Coverage | 142.772 |
|  | Mean Length | 16122.2 |
|  | N50 | 26897 |
|  | Read Number | 5868634 |
| Corrected reads | Base | 29417082519 |
|  | Coverage | 42.024 |
|  | Mean Length | 18814.133 |
|  | N50 | 27755 |
|  | Read Number | 1563563 |
|  | Yield from raw reads | 0.84 |
| Contigs | Number | 269 |
|  | Max Length | 29340420 |
|  | Mean Length | 2520673 |
|  | Median Length | 163815 |
|  | Min Length | 22587 |
|  | N50 | 23101701 |
|  | N90 | 2576042 |
|  | Total Base | 678061266 |

**Table S4.** Hi-C reads assessment of the genome assembly of the golden pompano.

| Item | Base | Percentage |
| --- | --- | --- |
| Sequenced Read Pairs | 382,798,592 | 100% |
| Normal Paired | 164,818,839 | 43.06% |
| Chimeric Paired | 181,192,725 | 47.33% |
| Chimeric Ambiguous | 30,086,194 | 7.86% |
| Unmapped | 6,700,834 | 1.75% |
| Ligation Motif Present | 250,420,851 | 65.42% |
| Unique Reads | 211,796,810 | 55.33% |
| PCR Duplicates: | 131,102,726 | 34.25% |
| Optical Duplicates | 3,112,028 | 0.81% |
| Intra-fragment Reads | 11,122,536 | 2.91% |
| Below MAPQ Threshold (30) | 34,920,632 | 9.12% |
| Hi-C Contacts | 165,753,642 | 43.30% |
| Ligation Motif Present | 75,940,491 | 19.84% |
| Pair Type %(L-I-O-R) |  | 25% - 25% - 25% - 25% |
| Inter-chromosomal | 90,263,953 | 23.58% |
| Intra-chromosomal | 75,489,689 | 19.72% |
| Short Range (<20Kb) | 52,443,660 | 13.70% |
| Long Range (>20Kb) | 23,045,547 | 6.02% |

**Table S5**. The statistics of genome assembly.

| Assembly | Primary contigs | Final scaffold |
| --- | --- | --- |
| Assembly size (bp) | 676174089 | 662700757 |
| Number of contigs | 245 |  |
| Longest contig size (bp) | 29354438 |  |
| Smallest contig size (bp) | 22937 |  |
| Contig N50 size (bp) | 23111360 |  |
| Number of scaffolds |  | 76 |
| Longest scaffold size (bp) |  | 33540323 |
| Smallest scaffold size (bp) |  | 5000 |
| Scaffold N50 size (bp) |  | 28467530 |
| Number of gaps |  | 32 |
| Length of gaps (bp) |  | 12812 |
| Bases in the 24 largest scaffolds (bp) |  | 651835813 |
| Bases in the 24 largest scaffolds (%) |  | 98.4 |

**Table S6**. BUSCOs of the genome assembly.

| Items | the golden pompano | Florida pompano |
| --- | --- | --- |
| Complete BUSCOs | 3559 | 3507 |
| Complete and single-copy BUSCOs | 3529 | 3469 |
| Complete and duplicated BUSCOs | 30 | 38 |
| Fragmented BUSCOs | 23 | 19 |
| Missing BUSCOs | 58 | 114 |
| Total BUSCO groups searched | 3640 | |

**Table S7**. Summary of chromosome-level genome assembly of the golden pompano.

| Serial Number | Scaffold | Linkage Group | Length (bp) | Genetic Distance (cM) | #Unique Loci | #SNPs in common | Pearson Correlation Coefficient | |
| --- | --- | --- | --- | --- | --- | --- | --- | --- |
| 1 | Chr1 | LG2 | 33540323 | 96.510 | 154 | 35169 | | 0.98 |
| 2 | Chr2 | LG17 | 32093560 | 82.509 | 127 | 25110 | | 0.98 |
| 3 | Chr3 | LG1 | 31242174 | 68.004 | 180 | 37875 | | 0.97 |
| 4 | Chr4 | LG4 | 30379813 | 81.005 | 154 | 34848 | | 0.98 |
| 5 | Chr5 | LG8 | 30111125 | 87.539 | 153 | 31155 | | 0.98 |
| 6 | Chr6 | LG13 | 30037430 | 97.527 | 155 | 29478 | | 0.99 |
| 7 | Chr7 | LG7 | 30037242 | 74.504 | 140 | 31256 | | 0.99 |
| 8 | Chr8 | LG12 | 29731833 | 81.005 | 149 | 30518 | | 0.97 |
| 9 | Chr9 | LG9 | 29483433 | 80.003 | 158 | 31020 | | 0.99 |
| 10 | Chr10 | LG6 | 29344570 | 100.296 | 136 | 31450 | | 0.99 |
| 11 | Chr11 | LG14 | 28467530 | 93.564 | 138 | 28738 | | 0.99 |
| 12 | Chr12 | LG5 | 27945398 | 81.509 | 148 | 32370 | | 0.99 |
| 13 | Chr13 | LG21 | 27478853 | 84.007 | 138 | 23108 | | 0.98 |
| 14 | Chr14 | LG3 | 26974502 | 94.689 | 128 | 35111 | | 0.95 |
| 15 | Chr15 | LG23 | 26583836 | 102.693 | 117 | 21947 | | 0.98 |
| 16 | Chr16 | LG11 | 26486104 | 74.527 | 143 | 30632 | | 0.98 |
| 17 | Chr17 | LG10 | 25698542 | 84.278 | 144 | 30976 | | 0.99 |
| 18 | Chr18 | LG20 | 24857111 | 69.505 | 113 | 24022 | | 0.98 |
| 19 | Chr19 | LG18 | 24202996 | 68.525 | 131 | 25027 | | 0.99 |
| 20 | Chr20 | LG19 | 23075506 | 71.087 | 123 | 24453 | | 0.99 |
| 21 | Chr21 | LG16 | 22605574 | 93.154 | 123 | 26431 | | 0.98 |
| 22 | Chr22 | LG15 | 21078717 | 77.774 | 134 | 26729 | | 0.97 |
| 23 | Chr23 | LG22 | 20660051 | 66.007 | 119 | 23088 | | 0.96 |
| 24 | Chr24 | LG24 | 19719590 | 74.180 | 102 | 18559 | | 0.88 |
| Total | - | - | 651835813 | 1984.401 | 3307 | 689070 | | - |
| Average | - | - | - | - | - | - | | 0.98 |

**Table S8**. The statistics of base scores of the final assembly, which was assessed by the software Referee (Thomas and Hahn 2019).

| Scaffold | Length(bp) | -2 | -1 | [0,30) | [30,60) | [60,90) | 90 | 91 |
| --- | --- | --- | --- | --- | --- | --- | --- | --- |
| Chr1 | 33540323 | 0.93% | 0.00% | 0.22% | 0.12% | 0.12% | 2.46% | 96.15% |
| Chr2 | 32093560 | 0.64% | 0.00% | 0.24% | 0.15% | 0.14% | 2.72% | 96.12% |
| Chr3 | 31242174 | 1.48% | 0.00% | 0.38% | 0.19% | 0.17% | 3.00% | 94.77% |
| Chr4 | 30379813 | 0.99% | 0.00% | 0.28% | 0.15% | 0.15% | 3.09% | 95.33% |
| Chr5 | 30111125 | 1.25% | 0.00% | 0.25% | 0.15% | 0.15% | 2.71% | 95.50% |
| Chr6 | 30037430 | 1.28% | 0.00% | 0.37% | 0.19% | 0.18% | 2.93% | 95.04% |
| Chr7 | 30037242 | 0.61% | 0.00% | 0.20% | 0.11% | 0.11% | 2.68% | 96.29% |
| Chr8 | 29731833 | 1.94% | 0.00% | 0.51% | 0.29% | 0.27% | 3.44% | 93.55% |
| Chr9 | 29483433 | 0.44% | 0.00% | 0.11% | 0.08% | 0.09% | 2.31% | 96.97% |
| Chr10 | 29344570 | 0.64% | 0.00% | 0.28% | 0.17% | 0.18% | 2.99% | 95.74% |
| Chr11 | 28467530 | 1.32% | 0.00% | 0.23% | 0.13% | 0.13% | 2.57% | 95.62% |
| Chr12 | 27945398 | 0.77% | 0.00% | 0.23% | 0.15% | 0.16% | 3.35% | 95.34% |
| Chr13 | 27478853 | 1.04% | 0.00% | 0.27% | 0.13% | 0.12% | 2.47% | 95.97% |
| Chr14 | 26974502 | 2.81% | 0.00% | 0.59% | 0.32% | 0.28% | 3.28% | 92.72% |
| Chr15 | 26583836 | 1.41% | 0.00% | 0.31% | 0.17% | 0.15% | 2.76% | 95.19% |
| Chr16 | 26486104 | 1.66% | 0.00% | 0.36% | 0.19% | 0.17% | 3.08% | 94.54% |
| Chr17 | 25698542 | 0.36% | 0.00% | 0.19% | 0.12% | 0.13% | 3.20% | 95.99% |
| Chr18 | 24857111 | 0.31% | 0.00% | 0.22% | 0.11% | 0.13% | 3.56% | 95.67% |
| Chr19 | 24202996 | 0.33% | 0.00% | 0.18% | 0.11% | 0.12% | 2.65% | 96.61% |
| Chr20 | 23075506 | 0.86% | 0.00% | 0.21% | 0.15% | 0.14% | 3.03% | 95.61% |
| Chr21 | 22605574 | 0.52% | 0.00% | 0.17% | 0.13% | 0.13% | 2.75% | 96.30% |
| Chr22 | 21078717 | 2.77% | 0.00% | 0.80% | 0.53% | 0.52% | 8.77% | 86.61% |
| Chr23 | 20660051 | 1.52% | 0.00% | 0.34% | 0.17% | 0.14% | 2.76% | 95.06% |
| Chr24 | 19719590 | 10.46% | 0.00% | 2.54% | 1.54% | 1.47% | 13.59% | 70.40% |
| unanchored contigs | 10864944 | 85.79% | 0.00% | 5.70% | 1.95% | 1.33% | 4.22% | 1.02% |

**Table S9**. Summary statistics of the annotated transposable elements.

| Repeat type | Number of elements | Length（bp） | Percentage in Genome (%) |
| --- | --- | --- | --- |
| Retroelements | 49686 | 13273613 | 2.00 |
| SINEs | 3973 | 485299 | 0.07 |
| Penelope | 722 | 66939 | 0.01 |
| LINEs | 30818 | 9757241 | 1.47 |
| L2/CR1/Rex | 21383 | 6697024 | 1.01 |
| R1/LOA/Jockey | 857 | 261143 | 0.04 |
| R2/R4/NeSL | 345 | 115647 | 0.02 |
| RTE/Bov-B | 2455 | 828812 | 0.13 |
| L1/CIN4 | 3730 | 1290881 | 0.19 |
| LTR elements | 14895 | 3031073 | 0.46 |
| BEL/Pao | 1217 | 542595 | 0.08 |
| Ty1/Copia | 52 | 73118 | 0.01 |
| Gypsy/DIRS1 | 7496 | 1682940 | 0.25 |
| Retroviral | 3186 | 401784 | 0.06 |
| DNA transposons | 101257 | 14167593 | 2.14 |
| hobo-Activator | 28527 | 2545655 | 0.38 |
| Tc1-IS630-Pogo | 17483 | 5862746 | 0.88 |
| PiggyBac | 959 | 141186 | 0.02 |
| Tourist/Harbinger | 6502 | 850334 | 0.13 |
| Other (Mirage, P-element, Transib) | 4418 | 682410 | 0.10 |
| Rolling-circles | 1213 | 154126 | 0.02 |
| Unclassified | 1025 | 208743 | 0.03 |
| Total interspersed repeats | | 27649949 | 4.17 |
| Small RNA | 5008 | 610678 | 0.09 |
| Satellites | 976 | 145203 | 0.02 |
| Simple repeats | 425453 | 18212714 | 2.75 |
| Low complexity | 50006 | 2670183 | 0.40 |

**Table S10**. Details of the protein-coding genes in pseudo-chromosomes.

| Chromosome | Gene Number | Average Length of the Genes |
| --- | --- | --- |
| Chr1 | 1044 | 11662.00 |
| Chr2 | 1235 | 10673.40 |
| Chr3 | 1317 | 10529.40 |
| Chr4 | 1243 | 10802.50 |
| Chr5 | 1260 | 10281.30 |
| Chr6 | 1055 | 11599.40 |
| Chr7 | 1102 | 11169.50 |
| Chr8 | 1261 | 10649.30 |
| Chr9 | 1184 | 11020.40 |
| Chr10 | 964 | 9923.49 |
| Chr11 | 1009 | 11966.80 |
| Chr12 | 1257 | 10405.10 |
| Chr13 | 1035 | 10629.90 |
| Chr14 | 1100 | 10500.10 |
| Chr15 | 937 | 11750.00 |
| Chr16 | 1289 | 9316.79 |
| Chr17 | 1059 | 9917.23 |
| Chr18 | 949 | 11614.80 |
| Chr19 | 942 | 10953.50 |
| Chr20 | 766 | 11525.50 |
| Chr21 | 1004 | 9853.13 |
| Chr22 | 1034 | 8793.74 |
| Chr23 | 668 | 10949.00 |
| Chr24 | 851 | 10840.90 |
| Debris | 155 | 8271.45 |

**SupTab 11.** The statistics of protein-coding gene.

|  | Transcript | | | | | | | | | | Exon | | | | Intron | | | |
| --- | --- | --- | --- | --- | --- | --- | --- | --- | --- | --- | --- | --- | --- | --- | --- | --- | --- | --- |
|  | Count | Length (bp) | | | Coding Length (bp) | | | Exon Number | | | Count | Length (bp) | | | Count | Length (bp) | | |
|  |  | Average | Median | Total | Average | Median | Total | Average | Median | Total |  | Average | Median | Total |  | Average | Median | Total |
| *Astyanax mexicanus* | 26662 | 22655.06 | 10078 | 604029195 | 1562.46 | 1182 | 41658289 | 8.72 | 6 | 232375 | 232369 | 179.27 | 126 | 41657598 | 205712 | 2464.46 | 887 | 506968096 |
| *Danio rerio* | 25447 | 28426.84 | 12881 | 723377794 | 1623.98 | 1195 | 41325523 | 9.38 | 7 | 238613 | 237954 | 173.26 | 124 | 41227115 | 212611 | 2859.99 | 1067 | 608066361 |
| *Gasterosteus aculeatus* | 20095 | 15245.25 | 6368 | 306353292 | 1459.03 | 1077 | 29319128 | 12.72 | 9 | 255675 | 255317 | 114.72 | 100 | 29291165 | 234950 | 1179.06 | 284 | 277020551 |
| *Larimichthys crocea* | 19569 | 39707.73 | 20295 | 777040537 | 1562.50 | 1179 | 30576622 | 9.94 | 7 | 194592 | 194569 | 157.14 | 118 | 30574056 | 175021 | 3956.13 | 1737 | 692405823 |
| *Lates calcarifer* | 25058 | 12729.50 | 5641 | 318975705 | 1548.62 | 1179 | 38805285 | 9.20 | 7 | 230470 | 230427 | 168.39 | 126 | 38800673 | 205396 | 1179.36 | 305 | 242236731 |
| *Lepisosteus oculatus* | 18341 | 24452.71 | 11130 | 448487198 | 1693.87 | 1251 | 31067290 | 10.68 | 8 | 195819 | 195801 | 158.65 | 121 | 31063577 | 177465 | 2093.07 | 711 | 371446812 |
| *Mola mola* | 21397 | 12750.81 | 6163 | 272828982 | 1467.56 | 1131 | 31401380 | 9.15 | 7 | 195679 | 195675 | 160.48 | 127 | 31401002 | 174282 | 1385.27 | 376 | 241427602 |
| *Oreochromis niloticus* | 28142 | 17038.50 | 6220 | 479497365 | 1682.47 | 1281 | 47348119 | 8.67 | 6 | 243864 | 243854 | 194.16 | 128 | 47347023 | 215711 | 1830.07 | 340 | 394766697 |
| *Oryzias latipes* | 22121 | 17003.78 | 8007 | 376140704 | 1639.96 | 1272 | 36277454 | 9.29 | 7 | 205498 | 205497 | 176.53 | 126 | 36277245 | 183375 | 1596.74 | 303 | 292802000 |
| *Poecilia formosa* | 23615 | 16629.53 | 8201 | 392706330 | 1679.11 | 1269 | 39652177 | 10.50 | 8 | 248055 | 247978 | 159.87 | 122 | 39643719 | 224391 | 1338.44 | 353 | 300334450 |
| *Takifugu rubripes* | 21383 | 10293.06 | 4337 | 220096504 | 1632.37 | 1263 | 34905019 | 9.40 | 7 | 201022 | 201020 | 173.64 | 126 | 34904780 | 179635 | 885.20 | 160 | 159012048 |
| *Tetraodon nigroviridis* | 19602 | 6192.20 | 3194 | 121379415 | 1516.59 | 1146 | 29728130 | 10.52 | 8 | 206132 | 206129 | 144.22 | 119 | 29727726 | 186529 | 477.87 | 118 | 89136899 |
| *Xiphophorus maculatus* | 23753 | 17334.31 | 8133 | 411741950 | 1641.11 | 1254 | 38981234 | 9.41 | 7 | 223412 | 223412 | 174.48 | 126 | 38981234 | 199658 | 1607.95 | 452 | 321039257 |
| *Seriola dumerili* | 23278 | 15428.97 | 7393 | 359155552 | 1630.45 | 1236 | 37953507 | 9.67 | 7 | 225046 | 225030 | 168.65 | 126 | 37951675 | 225030 | 1390.46 | 374 | 280546647 |
| ***Trachinotus ovatus*** | 25720 | 10669.68 | 7363 | 274424098 | 1623.82 | 1197 | 41764674 | 9.56 | 7 | 245954 | 245934 | 169.81 | 125 | 41762050 | 220217 | 924.85 | 380 | 203668634 |

Note: these statistics all were based on the longest transcript of genes.

**Table S12**. BUSCO of the transcripts used in phylogenetics. Total BUSCO groups is 3640.

| Species | Accession | Complete BUSCOs | Complete and single-copy BUSCOs | Complete and duplicated BUSCOs | Fragmented BUSCOs | Missing BUSCOs |
| --- | --- | --- | --- | --- | --- | --- |
| *Astyanax mexicanus* | Astyanax_mexicanus-2.0 | 93.6% | 3362 | 44 | 77 | 157 |
| *Danio rerio* | GRCz11 | 92.2% | 2760 | 597 | 51 | 232 |
| *Gasterosteus aculeatus* | BROADS1 | 87.4% | 3133 | 46 | 146 | 315 |
| *Larimichthys crocea* | L_crocea_2.0 | 95.4% | 3447 | 27 | 39 | 127 |
| *Lates calcarifer* | ASB_HGAPassembly_v1 | 91.7% | 3210 | 126 | 117 | 187 |
| *Lepisosteus oculatus* | LepOcu1 | 90.3% | 3238 | 46 | 103 | 253 |
| *Mola mola* | ASM169857v1 | 85.9% | 3064 | 62 | 125 | 389 |
| *Oreochromis niloticus* | O_niloticus_UMD_NMBU | 95.4% | 3444 | 29 | 43 | 124 |
| *Oryzias latipes* | ASM223467v1 | 91.3% | 3294 | 30 | 43 | 273 |
| *Poecilia formosa* | PoeFor_5.1.2 | 95.7% | 3352 | 131 | 55 | 102 |
| *Takifugu rubripes* | fTakRub1.2 | 90.8% | 3207 | 99 | 61 | 273 |
| *Tetraodon nigroviridis* | TETRAODON8 | 81.3% | 2885 | 74 | 185 | 496 |
| *Xiphophorus maculatus* | X_maculatus-5.0-mal | 95.7% | 3119 | 46 | 88 | 387 |
| *Seriola dumerili* | Seriola_dumerili.Sdu_1.0 | 96.7% | 3495 | 27 | 35 | 83 |
| ***Trachinotus ovatus*** |  | 87.6% | 3269 | 52 | 112 | 207 |

**Table S13**. Functional annotation of predicted protein-coding genes of the golden pompano.

| Item | Gene Number | Percentage |
| --- | --- | --- |
| Total | 25720 | 100% |
| Annotated | 24177 | 94.00% |
| GO Assigned | 16622 | 64.63% |
| KEGG_ko Assigned | 17463 | 67.90% |
| GO and KEGG_ko Both Assigned | 13180 | 51.24% |
| Preferred Name Assigned | 19213 | 74.70% |

**Table S14**. Summary of non-coding RNA in the annotation of the golden pompano*.*

| Item | Number of Type | Number of Copy | Alignment length (bp) |
| --- | --- | --- | --- |
| miRNA | 93 | 961 | 83140 |
| rRNA | 4 | 431 | 102397 |
| tRNA | - | 1155 | 85946 |
| cis-regulatory element | 10 | 409 | 22418 |
| snRNA | 16 | 356 | 53352 |
| snoRNA | 104 | 188 | 21702 |
| lncRNA | 4 | 4 | 665 |

**Table S15**. The primary transcripts used in phylogenetic trees construction.

| Species | Accession | Total Number | Discarded Number | Remained Number |
| --- | --- | --- | --- | --- |
| *Astyanax mexicanus* | Astyanax_mexicanus-2.0 | 26698 | 93 | 26605 |
| *Danio rerio* | GRCz11 | 30313 | 1859 | 28454 |
| *Gasterosteus aculeatus* | BROADS1 | 20787 | 147 | 20640 |
| *Larimichthys crocea* | L_crocea_2.0 | 22929 | 61 | 22868 |
| *Lates calcarifer* | ASB_HGAPassembly_v1 | 25109 | 4 | 25105 |
| *Lepisosteus oculatus* | LepOcu1 | 18341 | 348 | 17993 |
| *Mola mola* | ASM169857v1 | 21404 | 981 | 20423 |
| *Oreochromis niloticus* | O_niloticus_UMD_NMBU | 28189 | 27 | 28162 |
| *Oryzias latipes* | ASM223467v1 | 23622 | 53 | 23569 |
| *Poecilia formosa* | PoeFor_5.1.2 | 23615 | 175 | 23440 |
| *Takifugu rubripes* | fTakRub1.2 | 21411 | 10 | 21401 |
| *Tetraodon nigroviridis* | TETRAODON8 | 19602 | 307 | 19295 |
| *Xiphophorus maculatus* | X_maculatus-5.0-mal | 23774 | 52 | 23722 |
| *Seriola dumerili* | Seriola_dumerili.Sdu_1.0 | 23278 | 99 | 23179 |
| ***Trachinotus ovatus*** |  | 25720 | 0 | 25720 |
| Total |  | 331514 | 4160 | 327397 |

**SupTab 16**. The overall statistics of orthogroup assignment.

| Item | Number |
| --- | --- |
| Number of genes | 349091 |
| Number of genes in orthogroups | 331563 |
| Number of unassigned genes | 17528 |
| Percentage of genes in orthogroups | 95.0 |
| Percentage of unassigned genes | 5.0 |
| Number of orthogroups | 19804 |
| Number of species-specific orthogroups | 135 |
| Number of genes in species-specific orthogroups | 702 |
| Percentage of genes in species-specific orthogroups | 0.2 |
| Mean orthogroup size | 16.7 |
| Median orthogroup size | 15.0 |
| G50 (assigned genes) | 16 |
| G50 (all genes) | 16 |
| O50 (assigned genes) | 5731 |
| O50 (all genes) | 6278 |
| Number of orthogroups with all species present | 8475 |
| Number of single-copy orthogroups | 3093 |

**Table S17**. The statistics of orthogroups in each species.

|  | Number of genes | Number of species-specific orthogroups | Genes in orthogroups | | Unassigned genes | | Orthogroups containing species | | Genes in species-specific orthogroups | |
| --- | --- | --- | --- | --- | --- | --- | --- | --- | --- | --- |
|  |  |  | Number | Percentage | Number | Percentage | Number | Percentage | Number | Percentage |
| *Astyanax mexicanus* | 26605 | 21 | 24299 | 91.3 | 2306 | 8.7 | 16585 | 83.7 | 119 | 0.4 |
| *Danio rerio* | 28454 | 37 | 26307 | 92.5 | 2147 | 7.5 | 15772 | 79.6 | 255 | 0.9 |
| *Gasterosteus aculeatus* | 20640 | 6 | 19482 | 94.4 | 1158 | 5.6 | 15108 | 76.3 | 39 | 0.2 |
| *Larimichthys crocea* | 22868 | 0 | 22173 | 97.0 | 695 | 3.0 | 16674 | 84.2 | 0 | 0.0 |
| *Lates calcarifer* | 25105 | 2 | 24365 | 97.1 | 740 | 2.9 | 16810 | 84.9 | 4 | 0.0 |
| *Lepisosteus oculatus* | 17993 | 10 | 17079 | 94.9 | 914 | 5.1 | 14567 | 73.6 | 29 | 0.2 |
| *Mola mola* | 20423 | 1 | 19729 | 96.6 | 694 | 3.4 | 15476 | 78.1 | 2 | 0.0 |
| *Oreochromis niloticus* | 28162 | 20 | 26355 | 93.6 | 1807 | 6.4 | 16648 | 84.1 | 102 | 0.4 |
| *Oryzias latipes* | 22084 | 8 | 21171 | 95.9 | 913 | 4.1 | 15740 | 79.5 | 23 | 0.1 |
| *Poecilia formosa* | 23440 | 3 | 23113 | 98.6 | 327 | 1.4 | 16519 | 83.4 | 18 | 0.1 |
| *Seriola dumerili* | 23179 | 1 | 22679 | 97.8 | 500 | 2.2 | 16965 | 85.7 | 4 | 0.0 |
| *Takifugu rubripes* | 21401 | 6 | 20501 | 95.8 | 900 | 4.2 | 15355 | 77.5 | 30 | 0.1 |
| *Tetraodon nigroviridis* | 19295 | 5 | 18295 | 94.8 | 1000 | 5.2 | 14317 | 72.3 | 18 | 0.1 |
| ***Trachinotus ovatus*** | 25720 | 12 | 23063 | 89.7 | 2657 | 10.3 | 17001 | 85.8 | 50 | 0.2 |
| *Xiphophorus maculatus* | 23722 | 3 | 22952 | 96.8 | 770 | 3.2 | 16693 | 84.3 | 9 | 0.0 |

**Table S18**. Number of orthogroup in each catalog of orthogroup.

| Number of genes in orthogroup | 0 | 1 | 2 | 3 | 4 | 5 | 6 | 7 | 8 | 9 | 10 | 11-20 | 21-50 | 51+ |
| --- | --- | --- | --- | --- | --- | --- | --- | --- | --- | --- | --- | --- | --- | --- |
| *Astyanax mexicanus* | 3219 | 12603 | 3019 | 471 | 168 | 90 | 56 | 30 | 24 | 17 | 15 | 53 | 35 | 4 |
| *Danio rerio* | 4032 | 10187 | 3684 | 1158 | 348 | 160 | 65 | 30 | 19 | 21 | 7 | 60 | 29 | 4 |
| *Gasterosteus aculeatus* | 4696 | 12226 | 2335 | 294 | 113 | 47 | 29 | 13 | 10 | 8 | 4 | 21 | 6 | 2 |
| *Larimichthys crocea* | 3130 | 13137 | 2816 | 366 | 162 | 72 | 33 | 15 | 15 | 12 | 12 | 25 | 9 | 0 |
| *Lates calcarifer* | 2994 | 12578 | 3118 | 630 | 195 | 90 | 53 | 27 | 26 | 14 | 7 | 41 | 30 | 1 |
| *Lepisosteus oculatus* | 5237 | 13129 | 1093 | 195 | 58 | 27 | 14 | 15 | 8 | 3 | 0 | 16 | 8 | 1 |
| *Mola mola* | 4328 | 12466 | 2420 | 350 | 134 | 38 | 25 | 15 | 6 | 6 | 6 | 8 | 1 | 1 |
| *Oreochromis niloticus* | 3156 | 12950 | 2709 | 379 | 196 | 99 | 54 | 42 | 22 | 21 | 17 | 91 | 49 | 19 |
| *Oryzias latipes* | 4064 | 12771 | 2308 | 321 | 124 | 60 | 36 | 22 | 15 | 13 | 8 | 41 | 19 | 2 |
| *Poecilia formosa* | 3285 | 12692 | 2869 | 525 | 177 | 81 | 53 | 21 | 17 | 10 | 15 | 40 | 18 | 1 |
| *Seriola dumerili* | 2839 | 13343 | 2868 | 374 | 178 | 76 | 32 | 21 | 13 | 11 | 6 | 30 | 13 | 0 |
| *Takifugu rubripes* | 4449 | 12194 | 2459 | 399 | 129 | 59 | 31 | 20 | 15 | 5 | 10 | 21 | 12 | 1 |
| *Tetraodon nigroviridis* | 5487 | 11386 | 2350 | 382 | 103 | 46 | 17 | 6 | 11 | 2 | 6 | 8 | 0 | 0 |
| ***Trachinotus ovatus*** | 2803 | 13338 | 2792 | 482 | 175 | 76 | 42 | 21 | 13 | 12 | 4 | 34 | 10 | 2 |
| *Xiphophorus maculatus* | 3111 | 13282 | 2627 | 362 | 152 | 79 | 41 | 28 | 23 | 16 | 5 | 51 | 24 | 1 |

**Table S19**. Number of genes in each catalog of orthogroup.

| Number of genes  in orthogroup | 1 | 2 | 3 | 4 | 5 | 6 | 7 | 8 | 9 | 10 | 11-15 | 16-20 | 21-50 | 51-100 | 101-150 | 151+ |
| --- | --- | --- | --- | --- | --- | --- | --- | --- | --- | --- | --- | --- | --- | --- | --- | --- |
| *Astyanax mexicanus* | 12603 | 6038 | 1413 | 672 | 450 | 336 | 210 | 192 | 153 | 150 | 406 | 359 | 1061 | 256 | 0 | 0 |
| *Danio rerio* | 10187 | 7368 | 3474 | 1392 | 800 | 390 | 210 | 152 | 189 | 70 | 568 | 290 | 906 | 311 | 0 | 0 |
| *Gasterosteus aculeatus* | 12226 | 4670 | 882 | 452 | 235 | 174 | 91 | 80 | 72 | 40 | 241 | 34 | 161 | 124 | 0 | 0 |
| *Larimichthys crocea* | 13137 | 5632 | 1098 | 648 | 360 | 198 | 105 | 120 | 108 | 120 | 209 | 142 | 296 | 0 | 0 | 0 |
| *Lates calcarifer* | 12578 | 6236 | 1890 | 780 | 450 | 318 | 189 | 208 | 126 | 70 | 377 | 181 | 882 | 80 | 0 | 0 |
| *Lepisosteus oculatus* | 13129 | 2186 | 585 | 232 | 135 | 84 | 105 | 64 | 27 | 0 | 122 | 109 | 236 | 65 | 0 | 0 |
| *Mola mola* | 12466 | 4840 | 1050 | 536 | 190 | 150 | 105 | 48 | 54 | 60 | 48 | 68 | 22 | 92 | 0 | 0 |
| *Oreochromis niloticus* | 12950 | 5418 | 1137 | 784 | 495 | 324 | 294 | 176 | 189 | 170 | 706 | 623 | 1485 | 902 | 458 | 244 |
| *Oryzias latipes* | 12771 | 4616 | 963 | 496 | 300 | 216 | 154 | 120 | 117 | 80 | 320 | 278 | 586 | 154 | 0 | 0 |
| *Poecilia formosa* | 12692 | 5738 | 1575 | 708 | 405 | 318 | 147 | 136 | 90 | 150 | 325 | 230 | 545 | 54 | 0 | 0 |
| *Seriola dumerili* | 13343 | 5736 | 1122 | 712 | 380 | 192 | 147 | 104 | 99 | 60 | 304 | 108 | 372 | 0 | 0 | 0 |
| *Takifugu rubripes* | 12194 | 4918 | 1197 | 516 | 295 | 186 | 140 | 120 | 45 | 100 | 157 | 142 | 395 | 96 | 0 | 0 |
| *Tetraodon nigroviridis* | 11386 | 4700 | 1146 | 412 | 230 | 102 | 42 | 88 | 18 | 60 | 94 | 17 | 0 | 0 | 0 | 0 |
| ***Trachinotus ovatus*** | 13338 | 5584 | 1446 | 700 | 380 | 252 | 147 | 104 | 108 | 40 | 272 | 212 | 296 | 70 | 114 | 0 |
| *Xiphophorus maculatus* | 13282 | 5254 | 1086 | 608 | 395 | 246 | 196 | 184 | 144 | 50 | 457 | 292 | 698 | 60 | 0 | 0 |

**Table S20.** The data used for validating alternative splicing in gene *Hsd17b1*.

| Platform | Sex | Reads/Read Pairs (M) | Bases (G) | SRA ID |
| --- | --- | --- | --- | --- |
| Pacbio | Female | 10.6 | 15.7 | SRR14553106 |
|  | Male | 8.4 | 14.7 | SRR14553104 |
| Illumina | Female | 25.1 | 7.5 | SRR6168954 |
|  | Female | 43.7 | 13.1 | SRR14553107 |
|  | Female | 19.9 | 5.06 | SRR14553103 |
|  | Male | 21.4 | 6.4 | SRR6168955 |
|  | Male | 38.4 | 11.5 | SRR14553105 |
|  | Male | 17.3 | 4.3 | SRR14553102 |

**Table S21**. The sequences of *Hsd17b1*.

| Serial Number | Nucleotide | Protein | Species |
| --- | --- | --- | --- |
| 1 | / | BBE52722.1 | *Seriola dumerili* |
| 2 | LC389892.1 | BBE52723.1 | *Seriola dumerili* |
| 3 | / | BBE52724.1 | *Seriola quinqueradiata* |
| 4 | LC389894.1 | BBE52725.1 | *Seriola quinqueradiata* |
| 5 | AY306005.1 | AAP74564.1 | *Danio rerio* |
| 6 | XM_002935908.5 | XP_002935954.1 | *Xenopus tropicalis* |
| 7 | XM_003964791.3 | XP_003964840.1 | *Takifugu rubripes* |
| 8 | XM_010739661.3 | XP_010737963.1 | *Larimichthys crocea* |
| 9 | XM_014204314.1 | XP_014059789.1 | *Salmo salar* |
| 10 | XM_006004259.2 | XP_006004321.1 | *Latimeria chalumnae* |
| 11 | AB002410.1 | BAA19567.1 | *Gallus gallus* |
| 12 | XM_010585622.1 | XP_010583924.1 | *Haliaeetus leucocephalus* |
| 13 | XM_026654781.2 | XP_026510566.1 | *Terrapene carolina* |
| 14 | XM_006260403.3 | KYO33368.1 | *Alligator mississippiensis* |
| 15 | NM_010475.2 | NP_034605.1 | *Mus musculus* |
| 16 | NM_000413.4 | NP_000404.2 | *Homo sapiens* |

**Table S22**. Codon-based Test of Neutrality for analysis between sequences. Upper right matrix: test statistic (dN - dS), lower left matrix: *p* value.

|  |  | 1 | 2 | 3 | 4 | 5 | 6 | 7 | 8 | 9 | 10 | 11 | 12 | 13 | 14 | 15 |
| --- | --- | --- | --- | --- | --- | --- | --- | --- | --- | --- | --- | --- | --- | --- | --- | --- |
| 1 | *Seriola dumerili* |  | -3.07 | -5.77 | -8.19 | -12.34 | -11.48 | -13.54 | -10.88 | -13.47 | -9.46 | -9.96 | -14.29 | -10.90 | -8.74 | -10.98 |
| 2 | *Seriola quinqueradiata* | 0.00 |  | -5.53 | -8.18 | -12.46 | -11.65 | -13.23 | -10.85 | -13.82 | -9.72 | -10.24 | -14.37 | -11.23 | -8.85 | -11.11 |
| 3 | ***Trachinotus ovatus*** | 0.00 | 0.00 |  | -7.23 | -11.75 | -10.05 | -13.67 | -12.85 | -13.72 | -9.29 | -9.53 | -12.79 | -11.16 | -8.73 | -9.68 |
| 4 | *Larimichthys crocea* | 0.00 | 0.00 | 0.00 |  | -11.17 | -11.62 | -14.76 | -10.79 | -13.71 | -10.40 | -11.88 | -12.61 | -11.70 | -10.56 | -12.34 |
| 5 | *Takifugu rubripes* | 0.00 | 0.00 | 0.00 | 0.00 |  | -13.64 | -15.42 | -10.98 | -14.46 | -8.73 | -8.36 | -12.27 | -11.29 | -10.30 | -9.17 |
| 6 | *Salmo salar* | 0.00 | 0.00 | 0.00 | 0.00 | 0.00 |  | -13.24 | -10.01 | -11.90 | -8.24 | -8.72 | -9.77 | -9.41 | -11.36 | -7.84 |
| 7 | *Danio rerio* | 0.00 | 0.00 | 0.00 | 0.00 | 0.00 | 0.00 |  | -13.15 | -14.28 | -10.45 | -10.26 | -10.89 | -12.20 | -10.93 | -9.73 |
| 8 | *Latimeria chalumnae* | 0.00 | 0.00 | 0.00 | 0.00 | 0.00 | 0.00 | 0.00 |  | -16.27 | -9.42 | -7.33 | -11.21 | -9.75 | -8.50 | -7.39 |
| 9 | *Xenopus tropicalis* | 0.00 | 0.00 | 0.00 | 0.00 | 0.00 | 0.00 | 0.00 | 0.00 |  | -12.63 | -10.95 | -14.42 | -14.00 | -10.97 | -11.02 |
| 10 | *Haliaeetus_leucocephalus* | 0.00 | 0.00 | 0.00 | 0.00 | 0.00 | 0.00 | 0.00 | 0.00 | 0.00 |  | -6.21 | -6.88 | -7.03 | -7.78 | -6.27 |
| 11 | *Gallus gallus* | 0.00 | 0.00 | 0.00 | 0.00 | 0.00 | 0.00 | 0.00 | 0.00 | 0.00 | 0.00 |  | -8.33 | -6.42 | -7.85 | -4.69 |
| 12 | *Alligator mississippiensis* | 0.00 | 0.00 | 0.00 | 0.00 | 0.00 | 0.00 | 0.00 | 0.00 | 0.00 | 0.00 | 0.00 |  | -7.71 | -10.13 | -6.64 |
| 13 | *Terrapene carolina* | 0.00 | 0.00 | 0.00 | 0.00 | 0.00 | 0.00 | 0.00 | 0.00 | 0.00 | 0.00 | 0.00 | 0.00 |  | -8.83 | -6.12 |
| 14 | *Mus musculus* | 0.00 | 0.00 | 0.00 | 0.00 | 0.00 | 0.00 | 0.00 | 0.00 | 0.00 | 0.00 | 0.00 | 0.00 | 0.00 |  | -10.44 |
| 15 | *Homo sapiens* | 0.00 | 0.00 | 0.00 | 0.00 | 0.00 | 0.00 | 0.00 | 0.00 | 0.00 | 0.00 | 0.00 | 0.00 | 0.00 | 0.00 |  |

**Table S23**. Examples of heterozygosity levels in fish genomes.

| Common Name | Scientific Name | Heterozygosity (%) | Method | Reference |
| --- | --- | --- | --- | --- |
| **Atlantic silverside (GA)** | Menidia menidia | **1.76** | GenomeScope | (Tigano *et al*. 2021) |
| **Atlantic silverside (CT)** | Menidia menidia | **1.67** | GenomeScope | (Tigano *et al*. 2021) |
| European sardine | Sardina pilchardus | 1.60–1.75 | GenomeScope | (Machado *et al*. 2018) |
| American eel | Anguilla rostrata | 1.5–1.6 | GenomeScope | (Jansen *et al*. 2017) |
| European eel | Anguilla anguilla | 1.48–1.59 | GenomeScope | (Jansen *et al*. 2017) |
| **Atlantic silverside (CT)** | Menidia menidia | **1.46** | **Variant calling** | (Tigano *et al*. 2021) |
| Pearlscale pygmy angelfish | Centropyge vrolikii | 1.36 | GenomeScope | (Fernandez-Silva *et al*. 2018) |
| **Atlantic silverside (GA)** | Menidia menidia | **1.32** | **Variant calling** | (Tigano *et al*. 2021) |
| Marine medaka | Oryzias melastigma | 1.19 | GenomeScope | (Kim *et al*. 2018) |
| Large yellow croaker | Larimichthys crocea | 1.06 | GenomeScope | (Mu *et al*. 2018) |
| Javafish medaka | Oryzias javanicus | 0.96 | GenomeScope | (Takehana *et al*. 2020) |
| **Greater amberjack** | **Seriola dumerili** | 0.65 | GenomeScope | (Sarropoulou *et al*. 2017) |
| Clownfish | Amphiprion ocellaris | 0.60 | GenomeScope | (Tan *et al*. 2018) |
| Hilsa shad | Tenualosa ilisha | 0.58–0.66 | GenomeScope | (Mollah *et al*. 2019) |
| Whitefish | Coregonus sp. “Balchen” | 0.44 | GenomeScope | (De-KayneZoller and Feulner 2020) |
| Corkwing wrasse | Symphodus melops | 0.40 | GenomeScope | (Mattingsdal *et al*. 2018) |
| Herring | Clupea harengus | 0.32 | Variant calling | (Martinez *et al*. 2016) |
| Coelacanth | Latimeria chalumnae | 0.28 | Variant calling | (Amemiya *et al*. 2013) |
| NA | Lucifuga gibarensis | 0.26 | GenomeScope | (Policarpo *et al*. 2021) |
| Eurasian perch | Perca fluviatilis | 0.24–0.28 | GenomeScope | (Ozerov *et al*. 2018) |
| Atlantic cod | Gadus morhua | 0.20 | Variant calling | (Star *et al*. 2011) |
| Big-eye mandarin Fish | Siniperca knerii | 0.16 | GenomeScope | (LuZhao and Li 2020) |
| Threespine stickleback | Gasteosteus aculeatus | 0.14 | Variant calling | (Jones *et al*. 2012) |
| Pikeperch | Sander lucioperca | 0.14 | GenomeScope | (Nguinkal *et al*. 2019) |
| **The golden pompano** | **Trachinotus ovatus** | 0.34 | GenomeScope | This study |
| **snubnose pompano** | **Trachinotus blochii** | 0.21 | GenomeScope | This study |
| **Florida pompano** | **Trachinotus carolinus** | 0.25 | GenomeScope | This study |

**Table S24.** The sequencing primers.

| Items | Primers (5’->3’) | Amplicon Size | Target |  |
| --- | --- | --- | --- | --- |
| Zbf-Hsd17b1-F1 | ACGGAGCACATAACTGCACA | 472bp | Exon1 | zebrafish |
| Zbf-Hsd17b1-R2 | GACTGACAAAAATTGAAGACAACAA |  |  |  |
| Zbf-Hsd17b1-F3 | GGACAGGACAGCATTAAACTGA | 929bp | Exon2, Exon3 |  |
| Zbf-Hsd17b1-R4 | CAGGATTCTGCCGTGTCTCT |  |  |  |
| Gdp-Hsd17b1-F | CTCAGCCTGGCTGTCCG | 1018/954 bp in Z/W-linked transcripts | Exon1-6 | The golden pompano |
| Gdp-Hsd17b1-R | CAGTCAGTCAGAGGGCAT |  |  |  |

**Table S25.** The fertility assessment of zebrafish.

| Individuals | Mating | Total Eggs | Dead Eggs | Fertility Rate（%） |
| --- | --- | --- | --- | --- |
| AB 1 | First time | 149 | 10 | 93.3 |
|  | Second time | 126 | 3 | 97.6 |
|  | Third time | 352 | 8 | 97.7 |
| AB 2 | First time | 250 | 60 | 76 |
|  | Second time | 81 | 4 | 95.1 |
|  | Third time | 208 | 12 | 94.2 |
| AB 3 | First time | 270 | 16 | 94.1 |
|  | Second time | 251 | 8 | 96.8 |
|  | Third time | 223 | 5 | 97.8 |
| *Hsd17b1*#-5bp 1 | First time | 131 | 29 | 77.9 |
|  | Second time | 130 | 1 | 99.2 |
|  | Third time | 32 | 5 | 84.4 |
| *Hsd17b1*#-5bp 2 | First time | / | / | / |
|  | Second time | 181 | 8 | 95.6 |
|  | Third time | 305 | 70 | 77.0 |
| *Hsd17b1*#-2+7bp 1 | First time | 388 | 23 | 94.1 |
|  | Second time | 168 | 9 | 94.6 |
|  | Third time | 179 | 56 | 68.7 |
| *Hsd17b1*#-2+7bp 2 | First time | 292 | 7 | 97.6 |
|  | Second time | 50 | 3 | 94.0 |
|  | Third time | 270 | 23 | 91.5 |
| *Hsd17b1*#-2+7bp 3 | First time | 377 | 304 | 19.4 |
|  | Second time | 114 | 48 | 57.9 |
|  | Third time | 594 | 509 | 14.3 |
| *Hsd17b1*#-2+7bp 4 | First time | 463 | 156 | 66.3 |
|  | Second time | 136 | 26 | 80.9 |
|  | Third time | 476 | 142 | 70.2 |

**References**

Altschul, S. F., T. L. Madden, A. A. Schaffer, J. Zhang, Z. Zhang, W. Miller, and D. J. Lipman. 1997. Gapped BLAST and PSI-BLAST: a new generation of protein database search programs. NUCLEIC ACIDS RESEARCH **25**:3389-3402.

Amemiya, C. T., J. Alföldi, A. P. Lee, S. Fan, H. Philippe, I. MacCallum, I. Braasch, T. Manousaki, I. Schneider, N. Rohner, C. Organ, D. Chalopin, J. J. Smith, M. Robinson, R. A. Dorrington, M. Gerdol, B. Aken, M. A. Biscotti, M. Barucca, D. Baurain, A. M. Berlin, G. L. Blatch, F. Buonocore, T. Burmester, M. S. Campbell, A. Canapa, J. P. Cannon, A. Christoffels, G. De Moro, A. L. Edkins, L. Fan, A. M. Fausto, N. Feiner, M. Forconi, J. Gamieldien, S. Gnerre, A. Gnirke, J. V. Goldstone, W. Haerty, M. E. Hahn, U. Hesse, S. Hoffmann, J. Johnson, S. I. Karchner, S. Kuraku, M. Lara, J. Z. Levin, G. W. Litman, E. Mauceli, T. Miyake, M. G. Mueller, D. R. Nelson, A. Nitsche, E. Olmo, T. Ota, A. Pallavicini, S. Panji, B. Picone, C. P. Ponting, S. J. Prohaska, D. Przybylski, N. R. Saha, V. Ravi, F. J. Ribeiro, T. Sauka-Spengler, G. Scapigliati, S. M. J. Searle, T. Sharpe, O. Simakov, P. F. Stadler, J. J. Stegeman, K. Sumiyama, D. Tabbaa, H. Tafer, J. Turner-Maier, P. van Heusden, S. White, L. Williams, M. Yandell, H. Brinkmann, J. Volff, C. J. Tabin, N. Shubin, M. Schartl, D. B. Jaffe, J. H. Postlethwait, B. Venkatesh, F. Di Palma, E. S. Lander, A. Meyer, and K. Lindblad-Toh. 2013. The African coelacanth genome provides insights into tetrapod evolution. NATURE **496**:311-316.

Apweiler, R., A. Bairoch, C. H. Wu, W. C. Barker, B. Boeckmann, S. Ferro, E. Gasteiger, H. Huang, R. Lopez, and M. Magrane. 2004. UniProt: the universal protein knowledgebase. NUCLEIC ACIDS RESEARCH **32**:D115-D119.

Benton, M. J., and P. C. Donoghue. 2007. Paleontological evidence to date the tree of life. MOLECULAR BIOLOGY AND EVOLUTION **24**:26-53.

Bolger, A. M.M. Lohse, and B. Usadel. 2014. Trimmomatic: a flexible trimmer for Illumina sequence data. BIOINFORMATICS **30**:2114-2120.

Cantarel, B. L., I. Korf, S. M. C. Robb, G. Parra, E. Ross, B. Moore, C. Holt, A. Sánchez Alvarado, and M. Yandell. 2008. MAKER: an easy-to-use annotation pipeline designed for emerging model organism genomes. GENOME RESEARCH **18**:188-196.

Chin, C. S., D. H. Alexander, P. Marks, A. A. Klammer, J. Drake, C. Heiner, A. Clum, A. Copeland, J. Huddleston, E. E. Eichler, S. W. Turner, and J. Korlach. 2013. Nonhybrid, finished microbial genome assemblies from long-read SMRT sequencing data. NATURE METHODS **10**:563-569.

Chin, C. S., P. Peluso, F. J. Sedlazeck, M. Nattestad, G. T. Concepcion, A. Clum, C. Dunn, R. O'Malley, R. Figueroa-Balderas, A. Morales-Cruz, G. R. Cramer, M. Delledonne, C. Luo, J. R. Ecker, D. Cantu, D. R. Rank, and M. C. Schatz. 2016. Phased diploid genome assembly with single-molecule real-time sequencing. NATURE METHODS **13**:1050-1054.

De-Kayne, R.S. Zoller, and P. Feulner. 2020. A de novo chromosome-level genome assembly of Coregonus sp. "Balchen": One representative of the Swiss Alpine whitefish radiation. Molecular Ecology Resources **20**:1093-1109.

Dos Reis, M., and Z. Yang. 2019. Bayesian Molecular Clock Dating Using Genome-Scale Datasets. Methods in molecular biology (Clifton, N.J.) **1910**:309-330.

Durand, N. C., M. S. Shamim, I. Machol, S. S. P. Rao, M. H. Huntley, E. S. Lander, and E. L. Aiden. 2016. Juicer Provides a One-Click System for Analyzing Loop-Resolution Hi-C Experiments. Cell Systems **3**:95-98.

Emms, D., and S. L. Kelly. 2019. OrthoFinder: phylogenetic orthology inference for comparative genomics. GENOME BIOLOGY **20**:238.

English, A. C., S. Richards, Y. Han, M. Wang, V. Vee, J. Qu, X. Qin, D. M. Muzny, J. G. Reid, K. C. Worley, and R. A. Gibbs. 2012. Mind the gap: upgrading genomes with Pacific Biosciences RS long-read sequencing technology. PLoS One **7**:e47768.

Fernandez-Silva, I., J. B. Henderson, L. A. Rocha, and W. B. Simison. 2018. Whole-genome assembly of the coral reef Pearlscale Pygmy Angelfish (*Centropyge vrolikii*). Sci Rep **8**:1498.

Flicek, P., M. R. Amode, D. Barrell, K. Beal, K. Billis, S. Brent, D. Carvalho-Silva, P. Clapham, G. Coates, and S. Fitzgerald. 2013. Ensembl 2014. NUCLEIC ACIDS RESEARCH **42**:D749-D755.

Gao, Ge, Guo, An-Yuan, Bo, Luo, Jingchu, Jin, Jinpu, and Zhang. 2015. GSDS 2.0: an upgraded gene feature visualization server. BIOINFORMATICS.

Giles, S., G. Xu, T. J. Near, and M. Friedman. 2017. Early members of 'living fossil' lineage imply later origin of modern ray-finned fishes. NATURE **549**:265-268.

Guo, L., J. Yang, B. Liu, N. Zhang, K. Zhu, H. Guo, Q. Ma, Y. Li, S. Jiang, and D. Zhang. 2021. Colinearity based sex-specific marker development in the golden pompano (*Trachinotus ovatus*). AQUACULTURE **544**:737044.

Guo, L., Q. Yang, J. W. Yang, N. Zhang, B. S. Liu, K. C. Zhu, H. Y. Guo, S. G. Jiang, and D. C. Zhang. 2020. MultiplexSSR: A pipeline for developing multiplex SSR‐PCR assays from resequencing data. Ecology and Evolution **10**:3055-3067.

Hubley, R., R. D. Finn, J. Clements, S. R. Eddy, T. A. Jones, W. Bao, A. F. Smit, and T. J. Wheeler. 2016. The Dfam database of repetitive DNA families. NUCLEIC ACIDS RESEARCH **44**:D81-D89.

Huerta-Cepas, J., K. Forslund, L. P. Coelho, D. Szklarczyk, L. J. Jensen, C. von Mering, and P. Bork. 2017. Fast Genome-Wide Functional Annotation through Orthology Assignment by eggNOG-Mapper. MOLECULAR BIOLOGY AND EVOLUTION **34**:2115-2122.

Jansen, H. J., M. Liem, S. A. Jong-Raadsen, S. Dufour, F. A. Weltzien, W. Swinkels, A. Koelewijn, A. P. Palstra, B. Pelster, H. P. Spaink, G. Thillart, R. P. Dirks, and C. V. Henkel. 2017. Rapid de novo assembly of the European eel genome from nanopore sequencing reads. Sci Rep **7**:7213.

Jones, F. C., M. G. Grabherr, Y. F. Chan, P. Russell, E. Mauceli, J. Johnson, R. Swofford, M. Pirun, M. C. Zody, S. White, E. Birney, S. Searle, J. Schmutz, J. Grimwood, M. C. Dickson, R. M. Myers, C. T. Miller, B. R. Summers, A. K. Knecht, S. D. Brady, H. Zhang, A. A. Pollen, T. Howes, C. Amemiya, J. Baldwin, T. Bloom, D. B. Jaffe, R. Nicol, J. Wilkinson, E. S. Lander, F. Di Palma, K. Lindblad-Toh, and D. M. Kingsley. 2012. The genomic basis of adaptive evolution in threespine sticklebacks. NATURE **484**:55-61.

Kalvari, I., E. P. Nawrocki, J. Argasinska, N. Quinones-Olvera, R. D. Finn, A. Bateman, and A. I. Petrov. 2018. Non-Coding RNA Analysis Using the Rfam Database. Current protocols in bioinformatics **62**:e51.

Keibler, E., and M. R. Brent. 2003. Eval: a software package for analysis of genome annotations. BMC BIOINFORMATICS **4**:50.

Kim, D.B. Langmead, and S. L. Salzberg. 2015. HISAT: a fast spliced aligner with low memory requirements. NATURE METHODS **12**:357-360.

Kim, D., J. M. Paggi, C. Park, C. Bennett, and S. L. Salzberg. 2019. Graph-based genome alignment and genotyping with HISAT2 and HISAT-genotype. NATURE BIOTECHNOLOGY **37**:907-915.

Kim, H. S., B. Y. Lee, J. Han, C. B. Jeong, D. S. Hwang, M. C. Lee, H. M. Kang, D. H. Kim, D. Lee, J. Kim, I. Y. Choi, and J. S. Lee. 2018. The genome of the marine medaka *Oryzias melastigma*. Molecular Ecology Resources **18**:656-665.

Kolbe, D. L., and S. R. Eddy. 2009. Local RNA structure alignment with incomplete sequence. BIOINFORMATICS **25**:1236-1243.

Korf, I. 2004. Gene finding in novel genomes. BMC BIOINFORMATICS **5**:59.

Kuraku, S., C. M. Zmasek, O. Nishimura, and K. Katoh. 2013. aLeaves facilitates on-demand exploration of metazoan gene family trees on MAFFT sequence alignment server with enhanced interactivity. NUCLEIC ACIDS RESEARCH **41**:W22-W28.

Li, H. 2013. Aligning sequence reads, clone sequences and assembly contigs with BWA-MEM. arXiv:1303.3997.

Li, H. 2018. Minimap2: pairwise alignment for nucleotide sequences. BIOINFORMATICS **34**:3094-3100.

Li, H., B. Handsaker, A. Wysoker, T. Fennell, J. Ruan, N. Homer, G. Marth, G. Abecasis, and R. Durbin. 2009. The Sequence Alignment/Map format and SAMtools. BIOINFORMATICS **25**:2078-2079.

Lu, L.J. Zhao, and C. Li. 2020. High-Quality Genome Assembly and Annotation of the Big-Eye Mandarin Fish (*Siniperca knerii*). G3 (Bethesda) **10**:877-880.

Machado, A. M., O. K. Torresen, N. Kabeya, A. Couto, B. Petersen, M. Felicio, P. F. Campos, E. Fonseca, N. Bandarra, M. Lopes-Marques, R. Ferraz, R. Ruivo, M. M. Fonseca, S. Jentoft, O. Monroig, F. R. Da, and C. L. C. 2018. "Out of the Can": A Draft Genome Assembly, Liver Transcriptome, and Nutrigenomics of the European Sardine, *Sardina pilchardus*. Genes (Basel) **9**.

Marçais, G., and C. Kingsford. 2011. A fast, lock-free approach for efficient parallel counting of occurrences of k-mers. BIOINFORMATICS **27**:764-770.

Martinez, B. A., S. Lamichhaney, G. Fan, N. Rafati, M. Pettersson, H. Zhang, J. Dainat, D. Ekman, M. Hoppner, P. Jern, M. Martin, B. Nystedt, X. Liu, W. Chen, X. Liang, C. Shi, Y. Fu, K. Ma, X. Zhan, C. Feng, U. Gustafson, C. J. Rubin, A. M. Sallman, M. Blass, M. Casini, A. Folkvord, L. Laikre, N. Ryman, L. S. Ming-Yuen, X. Xu, and L. Andersson. 2016. The genetic basis for ecological adaptation of the Atlantic herring revealed by genome sequencing. eLife **5**.

Matthews, B. J., O. Dudchenko, S. B. Kingan, S. Koren, I. Antoshechkin, J. E. Crawford, W. J. Glassford, M. Herre, S. N. Redmond, N. H. Rose, G. D. Weedall, Y. Wu, S. S. Batra, C. A. Brito-Sierra, S. D. Buckingham, C. L. Campbell, S. Chan, E. Cox, B. R. Evans, T. Fansiri, I. Filipović, A. Fontaine, A. Gloria-Soria, R. Hall, V. S. Joardar, A. K. Jones, R. G. G. Kay, V. K. Kodali, J. Lee, G. J. Lycett, S. N. Mitchell, J. Muehling, M. R. Murphy, A. D. Omer, F. A. Partridge, P. Peluso, A. P. Aiden, V. Ramasamy, G. Rašić, S. Roy, K. Saavedra-Rodriguez, S. Sharan, A. Sharma, M. L. Smith, J. Turner, A. M. Weakley, Z. Zhao, O. S. Akbari, W. C. Black, H. Cao, A. C. Darby, C. A. Hill, J. S. Johnston, T. D. Murphy, A. S. Raikhel, D. B. Sattelle, I. V. Sharakhov, B. J. White, L. Zhao, E. L. Aiden, R. S. Mann, L. Lambrechts, J. R. Powell, M. V. Sharakhova, Z. Tu, H. M. Robertson, C. S. McBride, A. R. Hastie, J. Korlach, D. E. Neafsey, A. M. Phillippy, and L. B. Vosshall. 2018. Improved reference genome of *Aedes aegypti* informs arbovirus vector control. NATURE **563**:501-507.

Mattingsdal, M., S. Jentoft, O. K. Torresen, H. Knutsen, M. M. Hansen, J. I. Robalo, Z. Zagrodzka, C. Andre, and E. B. Gonzalez. 2018. A continuous genome assembly of the corkwing wrasse (*Symphodus melops*). GENOMICS **110**:399-403.

McKenna, A., M. Hanna, E. Banks, A. Sivachenko, K. Cibulskis, A. Kernytsky, K. Garimella, D. Altshuler, S. Gabriel, M. Daly, and M. A. DePristo. 2010. The Genome Analysis Toolkit: A Map Reduce framework for analyzing next-generation DNA sequencing data. GENOME RESEARCH **20**:1297-1303.

Mollah, M., M. Khan, M. S. Islam, and M. S. Alam. 2019. First draft genome assembly and identification of SNPs from hilsa shad (*Tenualosa ilisha*) of the Bay of Bengal. F1000Res **8**:320.

Mu, Y., J. Huo, Y. Guan, D. Fan, X. Xiao, J. Wei, Q. Li, P. Mu, J. Ao, and X. Chen. 2018. An improved genome assembly for *Larimichthys crocea* reveals hepcidin gene expansion with diversified regulation and function. Commun Biol **1**:195.

Nguinkal, J. A., R. M. Brunner, M. Verleih, A. Rebl, R. L. de Los, N. Schafer, F. Hadlich, M. Stueken, D. Wittenburg, and T. Goldammer. 2019. The First Highly Contiguous Genome Assembly of Pike perch (*Sander lucioperca*), an Emerging Aquaculture Species in Europe. Genes (Basel) **10**.

Nguyen, L. T., H. A. Schmidt, A. von Haeseler, and B. Q. Minh. 2015. IQ-TREE: a fast and effective stochastic algorithm for estimating maximum-likelihood phylogenies. MOLECULAR BIOLOGY AND EVOLUTION **32**:268-274.

Ozerov, M. Y., F. Ahmad, R. Gross, L. Pukk, S. Kahar, V. Kisand, and A. Vasemagi. 2018. Highly Continuous Genome Assembly of Eurasian Perch (*Perca fluviatilis*) Using Linked-Read Sequencing. G3 (Bethesda) **8**:3737-3743.

Pertea, M., D. Kim, G. M. Pertea, J. T. Leek, and S. L. Salzberg. 2016. Transcript-level expression analysis of RNA-seq experiments with HISAT, StringTie and Ballgown. Nature Protocols **11**:1650-1667.

Policarpo, M., J. Fumey, P. Lafargeas, D. Naquin, C. Thermes, M. Naville, C. Dechaud, J. N. Volff, C. Cabau, C. Klopp, P. R. Moller, L. Bernatchez, E. Garcia-Machado, S. Retaux, and D. Casane. 2021. Contrasting Gene Decay in Subterranean Vertebrates: Insights from Cavefishes and Fossorial Mammals. MOLECULAR BIOLOGY AND EVOLUTION **38**:589-605.

Purcell, S., B. Neale, K. Todd-Brown, L. Thomas, M. A. R. Ferreira, D. Bender, J. Maller, P. Sklar, P. I. W. de Bakker, M. J. Daly, and P. C. Sham. 2007. PLINK: A Tool Set for Whole-Genome Association and Population-Based Linkage Analyses. The American Journal of Human Genetics **81**:559-575.

Puttick, M. N. 2019. MCMCtreeR: functions to prepare MCMCtree analyses and visualize posterior ages on trees. BIOINFORMATICS **35**:5321-5322.

Rastas, P. 2017. Lep-MAP3: robust linkage mapping even for low-coverage whole genome sequencing data. BIOINFORMATICS **33**:3726-3732.

Sarropoulou, E., A. Sundaram, E. Kaitetzidou, G. Kotoulas, G. D. Gilfillan, N. Papandroulakis, C. C. Mylonas, and A. Magoulas. 2017. Full genome survey and dynamics of gene expression in the greater amberjack *Seriola dumerili*. GigaScience **6**:1-13.

Sim O, F. A., R. M. Waterhouse, I. Panagiotis, E. V. Kriventseva, and E. M. Zdobnov. 2015. BUSCO: assessing genome assembly and annotation completeness with single-copy orthologs. BIOINFORMATICS **31**:3210-3212.

Stanke, M., O. Keller, I. Gunduz, A. Hayes, S. Waack, and B. Morgenstern. 2006. AUGUSTUS: ab initio prediction of alternative transcripts. NUCLEIC ACIDS RESEARCH **34**:W435-W439.

Star, B., A. J. Nederbragt, S. Jentoft, U. Grimholt, M. Malmstrom, T. F. Gregers, T. B. Rounge, J. Paulsen, M. H. Solbakken, A. Sharma, O. F. Wetten, A. Lanzen, R. Winer, J. Knight, J. H. Vogel, B. Aken, O. Andersen, K. Lagesen, A. Tooming-Klunderud, R. B. Edvardsen, K. G. Tina, M. Espelund, C. Nepal, C. Previti, B. O. Karlsen, T. Moum, M. Skage, P. R. Berg, T. Gjoen, H. Kuhl, J. Thorsen, K. Malde, R. Reinhardt, Du L, S. D. Johansen, S. Searle, S. Lien, F. Nilsen, I. Jonassen, S. W. Omholt, N. C. Stenseth, and K. S. Jakobsen. 2011. The genome sequence of Atlantic cod reveals a unique immune system. NATURE **477**:207-210.

Suyama, M.D. Torrents, and P. Bork. 2006. PAL2NAL: robust conversion of protein sequence alignments into the corresponding codon alignments. NUCLEIC ACIDS RESEARCH **34**:609-612.

Takehana, Y., M. Zahm, C. Cabau, C. Klopp, C. Roques, O. Bouchez, C. Donnadieu, C. Barrachina, L. Journot, M. Kawaguchi, S. Yasumasu, S. Ansai, K. Naruse, K. Inoue, C. Shinzato, M. Schartl, Y. Guiguen, and A. Herpin. 2020. Genome Sequence of the Euryhaline Javafish Medaka, Oryzias javanicus: A Small Aquarium Fish Model for Studies on Adaptation to Salinity. G3 (Bethesda) **10**:907-915.

Tan, M. H., C. M. Austin, M. P. Hammer, Y. P. Lee, L. J. Croft, and H. M. Gan. 2018. Finding Nemo: hybrid assembly with Oxford Nanopore and Illumina reads greatly improves the clownfish (*Amphiprion ocellaris*) genome assembly. GigaScience **7**:1-6.

Thomas, G. W. C., and M. W. Hahn. 2019. Referee: Reference Assembly Quality Scores. Genome Biology and Evolution **11**:1483-1486.

Tigano, A., A. Jacobs, A. P. Wilder, A. Nand, Y. Zhan, J. Dekker, and N. O. Therkildsen. 2021. Chromosome-Level Assembly of the Atlantic Silverside Genome Reveals Extreme Levels of Sequence Diversity and Structural Genetic Variation. Genome Biology and Evolution **13**.

Turner, S. D. 2014. qqman: an R package for visualizing GWAS results using Q-Q and manhattan plots. Biorxiv.

Vurture, G. W., F. J. Sedlazeck, M. Nattestad, C. J. Underwood, H. Fang, J. Gurtowski, and M. C. Schatz. 2017. GenomeScope: fast reference-free genome profiling from short reads. BIOINFORMATICS **33**:2202-2204.

Walker, B. J., T. Abeel, T. Shea, M. Priest, A. Abouelliel, S. Sakthikumar, C. A. Cuomo, Q. Zeng, J. Wortman, S. K. Young, and A. M. Earl. 2014. Pilon: an integrated tool for comprehensive microbial variant detection and genome assembly improvement. PLoS One **9**:e112963.

Wu, T. D., and C. K. Watanabe. 2005. GMAP: a genomic mapping and alignment program for mRNA and EST sequences. Bioinformatics (Oxford, England) **21**:1859-1875.

Yang, J., X. Chen, J. Bai, D. Fang, Y. Qiu, W. Jiang, H. Yuan, C. Bian, J. Lu, S. He, X. Pan, Y. Zhang, X. Wang, X. You, Y. Wang, Y. Sun, D. Mao, Y. Liu, G. Fan, H. Zhang, X. Chen, X. Zhang, L. Zheng, J. Wang, L. Cheng, J. Chen, Z. Ruan, J. Li, H. Yu, C. Peng, X. Ma, J. Xu, Y. He, Z. Xu, P. Xu, J. Wang, H. Yang, J. Wang, T. Whitten, X. Xu, and Q. Shi. 2016. The Sinocyclocheilus cavefish genome provides insights into cave adaptation. BMC BIOLOGY **14**:1.

Yang, Z. 2007. PAML 4: Phylogenetic Analysis by Maximum Likelihood. MOLECULAR BIOLOGY AND EVOLUTION **24**:1586-1591.

Zhang, D., L. Guo, H. Guo, K. Zhu, S. Li, Y. Zhang, N. Zhang, B. Liu, S. Jiang, and J. Li. 2019. Chromosome-level genome assembly of golden pompano (*Trachinotus ovatus*) in the family Carangidae. Scientific Data **6**:1-11.

Zou, C., L. Wang, Y. Zou, Z. Wu, W. Wang, S. Liang, L. Wang, and F. You. 2020. Characteristics and sex dimorphism of 17beta-hydroxysteroid dehydrogenase family genes in the olive flounder *Paralichthys olivaceus*. J Steroid Biochem Mol Biol **199**:105597.
